# Investigating treatment-effect modification by a continuous covariate in IPD meta-analysis: an approach using fractional polynomials

**DOI:** 10.1186/s12874-022-01516-w

**Published:** 2022-04-06

**Authors:** Willi Sauerbrei, Patrick Royston

**Affiliations:** 1 Institute of Medical Biometry and Statistics, Faculty of Medicine and Medical Center - University of Freiburg, Freiburg, Germany; 2 MRC Clinical Trials Unit at UCL, Institute of Clinical Trials and Methodology, University College London, London, UK

**Keywords:** Meta-analysis, Continuous covariate, Treatment-effect modification, Fractional polynomials, Structured reporting

## Abstract

**Background:**

In clinical trials, there is considerable interest in investigating whether a treatment effect is similar in all patients, or that one or more prognostic variables indicate a differential response to treatment. To examine this, a continuous predictor is usually categorised into groups according to one or more cutpoints. Several weaknesses of categorization are well known. To avoid the disadvantages of cutpoints and to retain full information, it is preferable to keep continuous variables continuous in the analysis. To handle this issue, the Subpopulation Treatment Effect Pattern Plot (STEPP) was proposed about two decades ago, followed by the multivariable fractional polynomial interaction (MFPI) approach. Provided individual patient data (IPD) from several studies are available, it is possible to investigate for treatment heterogeneity with meta-analysis techniques. Meta-STEPP was recently proposed and in patients with primary breast cancer an interaction of estrogen receptors with chemotherapy was investigated in eight randomized controlled trials (RCTs).

**Methods:**

We use data from eight randomized controlled trials in breast cancer to illustrate issues from two main tasks. The first task is to derive a treatment effect function (TEF), that is, a measure of the treatment effect on the continuous scale of the covariate in the individual studies. The second is to conduct a meta-analysis of the continuous TEFs from the eight studies by applying pointwise averaging to obtain a mean function. We denote the method metaTEF. To improve reporting of available data and all steps of the analysis we introduce a three-part profile called MethProf-MA.

**Results:**

Although there are considerable differences between the studies (populations with large differences in prognosis, sample size, effective sample size, length of follow up, proportion of patients with very low estrogen receptor values) our results provide clear evidence of an interaction, irrespective of the choice of the FP function and random or fixed effect models.

**Conclusions:**

In contrast to cutpoint-based analyses, metaTEF retains the full information from continuous covariates and avoids several critical issues when performing IPD meta-analyses of continuous effect modifiers in randomised trials. Early experience suggests it is a promising approach.

**Trial registration:**

Not applicable.

**Supplementary Information:**

The online version contains supplementary material available at 10.1186/s12874-022-01516-w.

## Background

Personalized, precision or stratified medicine are popular terms used interchangeably for the separation of patients into several subgroups according to biological or risk characteristics, with the potential that a ‘best’ intervention can be targeted to the individual patient. Defining suitable subgroups and determining which intervention is ‘best’ for each group is a challenging task requiring investigation of differential treatment effects. See [1] for a discussion of general issues and [2] for arguments that a factor potentially predicting differential treatment response should be evaluated in randomized trials. Nowadays, subgroup analyses are a routine part of clinical trials and graphical approaches play a key role in subgroup analyses to visualise effect sizes of subgroups, to aid the identification of groups that respond differentially, and to communicate the results to a wider audience. However, many existing approaches do not capture the core information and are prone to lead to a misinterpretation of the subgroup effects [3].

Presumably many proponents of stratified medicine (our preferred term) are unaware that evidence for the effectiveness of individualized therapies must come from investigations of ‘complex’ interactions in large randomized trials. Interactions between treatment and covariates, such as prognostic factors, in randomized trials are essential ingredients of individualized treatments to check ‘whether one size fits all’. In cancer studies, such factors are often called predictive. When the covariate is continuous (such as age or hormone receptor level), such interactions are often sought by crude statistical methods, typically categorizing the continuous covariate. Royston et al. discuss and illustrate several disadvantages of dichotomization and call it a ‘bad idea’ [4].

In paper 4 on stratified medicine research in the Prognosis Research Strategy (PROGRESS) series, the group made several recommendations to improve medical research [2]. Among others, they recommend ‘*Research to identify factors that truly predict treatment effect could be improved by … increasing statistical power … by analysing continuous factors on their original scale’*. In summary, recommendation 13 of the supplementary table reads ‘*Standards in statistical analyses of prognosis research should be developed which address the multiple current limitations. In particular, continuous variables should be analyzed on their continuous scale and non-linear relationships evaluated as appropriate’*.

To use the full information from continuous variables such as treatment-effect modifiers, Royston and Sauerbrei proposed the multivariable fractional polynomial interaction (MFPI) method [5], an extension of the multivariable fractional polynomial (MFP) procedure [6]. First, MFPI estimates for each treatment group a fractional polynomial function representing the prognostic effect of the continuous covariate in the treatment group. Second, the difference between the functions for the two (or more) treatment groups is calculated and tested for significance. A plot of the difference (eg, log hazard ratio) against the covariate, together with a 95% CI, is termed a “treatment-effect plot.” A treatment-effect plot for a continuous covariate not interacting with treatment would be a straight line parallel to the x-axis, whereas a treatment-covariate interaction would be indicated by an increasing or a decreasing line or curve. Optionally, estimates of treatment effects can be adjusted for other covariates. Using bootstrap methodology, (in)stability of functions can be investigated. In a specific example, instability of functions selected was more severe in the extremes with a small number of observations, but altogether it was no serious issue [7]. To allow more or less flexibility of the main effects and interaction models, several modifications of MFPI were proposed [8, 9] and several alternative approaches (e.g. replace FP function by functions based on categorization or splines) are available. To investigate type I error and power, Royston and Sauerbrei [9, 10] performed a simulation study of 21 methods of detecting and modelling interactions between a binary ‘treatment’ variable and a continuous ‘prognostic factor’, including splines and categorization. They concluded *‘We believe that the results provide sufficient evidence to recommend the multivariable fractional polynomial interaction procedure as a suitable approach to investigate interactions of treatment with a continuous variable. If subject-matter knowledge gives good arguments for a non-monotone treatment effect function, we propose to use a second-degree fractional polynomial approach, but otherwise a first-degree fractional polynomial (FP1) function with added flexibility (flex3) is the method of choice.’*

Individual participant data (IPD) meta-analysis of randomized trials provides perhaps the best method of investigating treatment-covariate interactions [11]. For a summary of continuous functions from several studies a meta-analysis approach was recently proposed [12] which can be applied to obtain a mean treatment-effect function. Originally developed for the assessment of continuous prognostic factors or risk factors, its adaptation to meta-analysis of treatment-effect functions from several randomized trials is straightforward [13].

The subpopulation treatment effect pattern plot (STEPP) is another approach to investigate interaction with a continuous variable [14–16]. Similarities to and differences from MFPI are discussed and illustrated in an example [8, 17]. Meta-STEPP, a meta-analysis version of STEPP, was recently proposed for the analysis of several studies [18]. For illustration, the authors used IPD from eight randomized trials in primary breast cancer to explore whether the effect of chemotherapy varies according to estrogen receptor values, measured quantitatively in fmol/mg cytosol protein, in patients treated with hormonal therapy.

We reuse the data here to illustrate metaTEF, our meta-analysis approach to treatment effect functions estimated using MFPI in the individual studies. For analyzing the latter, we use the proposed default FP1 (flex3) approach in MFPI whereby the functions are averaged in the same manner as for continuous prognostic factors [12]. White et al. termed the procedure ‘metacurve’ in a recent comparison of methods [19]. Through sensitivity analyses we illustrate various issues arising from our approach and some additional points raised by the specific example.

The interpretation of individual studies and of meta-analyses depends critically on patients included, methods used and on analyses conducted. In clinical trials it is good practice that analyses are laid out in a statistical analysis plan [20] but that is less common in observational studies. Often, several analyses are conducted and reported selectively, resulting in biased results and biased interpretation. The problem of overinterpretation and misreporting of prognostic factor studies has been demonstrated empirically, even in high impact journals [21, 22]. The REMARK profile [23] is a tool aimed at improving reporting while emphasizing completeness and clarity of all analyses conducted. Here we adapt the concept for the analysis steps in a meta-analysis. The PRISMA statement was proposed for the reporting of systematic reviews and meta-analyses [24], but our methodological paper is much different and it does not make sense to follow PRISMA. Therefore, we use the name ‘Methodological Profile – Meta-Analysis’ (‘MethProf-MA’).

In section “Example: Effect of chemotherapy in patients with breast cancer” we provide pertinent details of the eight RCTs. To give an overview of available data and the analysis strategy, we show all steps of the analysis in a three-part profile (MethProf-MA). The upper and middle part of Table 1 provide an overview of the general information about available data, including data description and results of main effect of chemotherapy in each study. The lower part provides an overview of all analyses conducted to investigate for interactions of treatment with a predictor and several related sensitivity analyses. In section “Methods” we describe estimation of the treatment effect function and the meta-analysis approach to averaging functions from several studies. Results of the investigations for interaction are presented in section “Results”. In section “Discussion” we discuss the key steps of our novel approach for the analysis of interactions with continuous variables and the results of the investigation for an interaction between chemotherapy and estrogen receptor in breast cancer patients treated with hormonal therapy.

**Table 1:** Methodological Profile - Meta-Analysis (MethProf-MA). Three part profile with (top) general information about available data, (middle) description of data and main effect of CT, (bottom) analyses to investigate interactions of treatment with a predictor

**A: General information about available data**		
**Data sources**	Breast Cancer DataMart (Provided by NCI) Contains IPD from 14 published NCI-sponsored breast cancer studies. 8 studies assessed effect of adding chemotherapy (CT-Y/N) to tamoxifen. Here we include only tamoxifen treated patients. For details see section 4 in Wang et al.	
**Study question**	Does effect of CT depend on estrogen receptor (ER) values?	
**Data**	CT-N: *N* = 2982, No ER = 401, in study 2581 CT-Y: *N* = 3586, No ER = 440, in study 3146 Outcome: disease free survival (DFS) Events: Ct-*N* = 1342, CT-Y=1601, overall. See Table 2 for more details. We use ER+1 (see “General issues of the three-stage metaTEF approach”) and we truncate all ER values at 1000, which made little difference (see “Estrogen receptor”).	
**Variables**	Treatment: CT (Y/N); Outcome: recurrence free survival (RFS); Predictor: ER	
**B: Description of data and main effect of CT**		
**D1 – per study**	Patients, Follow-Up, Events, *p*-values (CT-Y/N)	Table 2
**D2 – per study**	Estimates of DFS rates, combined for CT	Fig. 1
**D3 – per study**	Effect of CT in each study	Fig. A1, *p*-values in Table 2, check of PH assumption from the Cox model.
**D4: ER – per study**	Distribution	Table 3, Fig. A2, Estrogen receptor
**C: Analyses to investigate interactions of treatment with a predictor**		
**A1 – per study**	Interaction of CT with ER?	Table 2, Preliminary analyses
**A2 – ER subgroups** **studywise and pooled estimate**	Effect of CT in 4 ER subgroups	Table A2, Fig. A3, Preliminary analyses
**A3 – TEF – studywise and pooled estimate**	Treatment effect function (TEF) for log ER	Power 0 (log ER) in each study (see Fig. A4), Preliminary analyses
**A4 – studywise FP1**	TEF (FP1, flex3) separately per study	Fig. 2a, metaTEF
**A5 – metaTEF for A4**	Main analysis - Variances, weights per study and metaTEF	Fig. 2b – f
**A6 – studywise and pooled FP2**	As **A4** but with FP2 (flex1)	Fig. A5, Table A3 sensitivity analysis FP1 or FP2?
**A7 – metaTEF**	Comparison of FP1/FP2 and fixed/random effect	Fig. 3
**A8 – CI of metaTEF**	Pointwise 95% CI for FP1/FP2 and fixed/random effect	Fig. A6

The profile MethProf-MA, providing an overview of studies, patients, main effect of chemotherapy and conducted analyses to investigate interactions of chemotherapy with estrogen receptors

## Example: effect of chemotherapy in patients with breast cancer

Breast Cancer DataMart (BCDM) is a resource provided by the National Cancer Institute that contains IPD from 14 previously published National Cancer Institute-sponsored randomized breast cancer trials. Wang et al. applied Meta-STEPP to relevant arms (patients treated with tamoxifen) of eight trials in BCDM to explore whether the effect of chemotherapy changes with the level of estrogen receptor (ER) expression [18]. The data for an analysis with metaTEF were provided by Breast Cancer DataMart Consortium, which is funded by US NCI/CTEP. The authors of the Meta-STEPP paper helped us to clarify details. Like Wang et al. [18] we use disease free survival (DFS) time as our outcome and estimate the treatment effect by the log-hazard ratio of chemotherapy vs no chemotherapy. A brief overview of the patient population is given in Table 1. Further details about the definition of DFS, measurement techniques and more are given in Wang et al. [18] and cited references. As did Wang et al., we analyse the data from all patients according to the intention-to-treat principle. We substituted a small positive time of 0.001 months as the censoring times of 14 patients who were randomized but had no follow-up.

Table 1 provides an overview of available data, analyses to investigate for main effects in each study (Table 1, middle part) and analyses to investigate for interactions (Table 1, lower part). Results of the latter are presented in section “Results”.

### Number of patients and main effect of chemotherapy

**Fig. 1:**
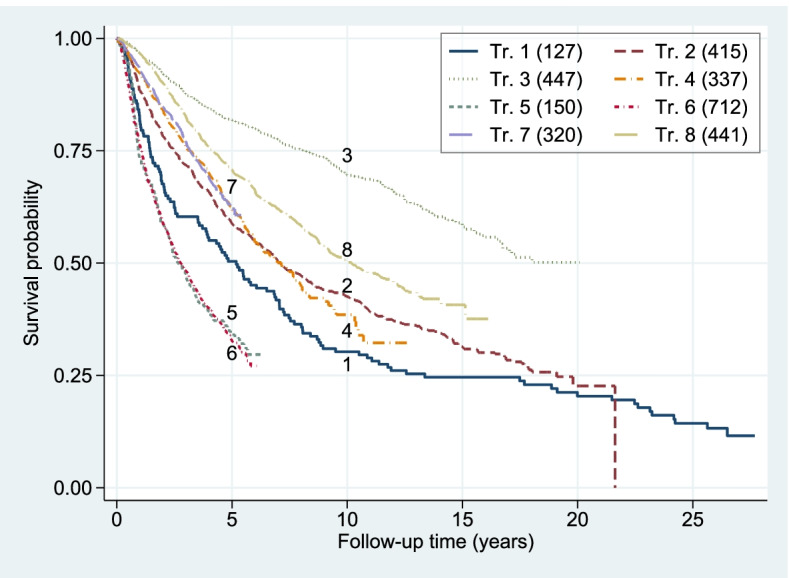
Disease-free survival by trial. Number of events in parentheses

In Table 2 and Fig. 1 we provide numbers of patients and events for each study and show DFS estimates. The patient populations differ substantially, with estimated 5-year DFS probabilities ranging from 0.82 to 0.33. Figure 1 also illustrates that maximum follow-up time ranges from about 5 years to nearly 30 years. In Fig. A1, we show DFS estimates for each study by treatment group. In the trial with the longest follow-up time (Trial 1) the proportional hazards assumption seems to be (slightly) violated. There is no strong indication of non-proportionality in any of the other trials. In what follows we assume that the PH assumption is acceptable for all trials.

**Table 2:** Number of patients and events per study. Percentage of events per treatment group and *p*-values from the test for interactions (FP1, flex3), overall (pooled data, stratified by trial) *p* = 0.0215

	Trial	Patients	Events		% events by treatment		*p*-values	*p*-values
			No	Yes	CT-N	CT-Y	CT	CT-ER Interact.
1	IBCSG-3	152	25	127	86.7%	80.5%	0.1519	0.0917
2	IBCSG-7	592	177	415	71.1%	69.1%	0.2089	0.8729
3	IBCSG-9	1177	730	447	38.7%	37.2%	0.2945	0.1198
4	NCIC-MA4	731	394	337	46.8%	45.4%	0.5505	0.2195
5	NSABP-B16–1	224	74	150	63.5%	68.7%	0.6368	0.2444
6	NSABP-B16–2	1071	359	712	72.2%	63.6%	0.0009	0.1027
7	NSABP-B20	966	646	320	34.7%	31.5%	0.3501	0.5155
8	SWOG-S8814	814	373	441	58.5%	51.2%	0.0320	0.0081

Only two of the eight studies (NSABP-BP16–2 and SWOG-S8814) showed a conventionally significant advantage of chemotherapy (*p* < 0.05) whereas the other six studies have *p*-values above 0.10 (Table 2). *p*-values were calculated from the Cox partial likelihood ratio test.

### Estrogen receptor

There is a lengthy debate concerning an interaction of hormonal treatment with estrogen receptor values [25, 26]. Much centers around the definition of receptor positivity, which depends on the chosen cutpoints if a continuous measurement was used for this classification. As in many breast cancer studies worldwide, estrogen receptors were measured quantitatively in fmol/mg cytosol protein (henceforth simply fmol) in the eight studies [18]. For many years, definitions concerning estrogen receptor positivity centered around values from 5 to 20 fmol, but more recently 1 fmol was discussed as the appropriate cutpoint [27, 28]. Clinically, the range 0 to 20 fmol is considered the most relevant. In Table 3 we provide details of this range in each of the studies. ER distributions are given in Fig. A2. Three studies (4, 7, 8) have only a small percentage (< 3%) of values up to 5 fmol, whereas other studies even have a substantial number of patients lacking any receptors. Extreme large values (> 1000 fmol) are rare (overall below 0.8%).

**Table 3:** Distribution of ER per study, categorized into 7 intervals. Given are percentages per interval. See Fig. A2 for more details

	ER (fmol/l)							
	0	1–5	6–10	11–20	21–100	101–1000	> 1000	Size
IBCSG-3	17.1	10.5	5.9	8.6	30.9	27.0	0.0	**152**
IBCSG-7	6.4	9.3	7.8	8.8	24.0	42.4	1.4	**592**
IBCSG-9	3.9	13.9	8.6	10.4	25.4	36.8	1.1	**1177**
NCIC-MA4	0.7	1.8	2.5	7.4	35.6	51.6	0.6	**731**
NSABP-B16–1	8.9	4.9	6.7	9.8	31.7	37.5	0.5	**224**
NSABP-B16–2	10.4	4.5	3.7	8.4	35.8	36.8	0.5	**1071**
NSABP-B20	0.1	0.0	1.0	12.4	42.9	42.6	1.0	**966**
SWOG-S8814	0.1	2.8	3.2	8.7	35.4	49.3	0.5	**814**
Total	4.3	5.7	4.6	9.5	33.3	41.8	0.8	**5727**

To circumvent potential problems caused by influential points, we truncated all ER values at 1000, meaning that values above 1000 fmol were replaced by the value 1000. In a sensitivity analysis we checked the influence of this decision. It made little difference (data not shown).

As the estimation of a treatment effect function with fractional polynomial methodology requires positive values for ER we use ER + 1 in the following (see “General issues of the three-stage metaTEF approach” for discussion).

## Methods

### Fractional polynomials and its variants in MFPI

The class of fractional polynomial (FP) functions is an extension of power transformations of a variable [29]. For most applications FP1 and FP2 functions are sufficient.

FP1: β_1_x^p1^, FP2: β_1_x^p1^ + β_2_x^p2^.

For the exponents p_1_ and p_2_ a set S with 8 values was proposed:

S = {− 2, − 1, − 0.5, 0, 0.5, 1, 2, 3}, where 0 means log x. For p_1_ = p_2_ = p (‘repeated powers’) it is defined

FP2 = β_1_x^p^ + β_2_x^p^ log x. This defines 8 FP1 and 36 FP2 models.

The MFPI algorithm was published in its original form by Royston and Sauerbrei [5]. Some modifications to increase the flexibility of MFPI were suggested later [8]. Depending on the nature of the study, the analyst must decide whether to use an FP1 or FP2 model with MFPI. For both FP1 and FP2 models, four levels of flexibility may be used (Table A1). The default is the original, least flexible algorithm, called flex1. Here, FP powers are determined for the main effect of the continuous covariate x in a model excluding a t by x interaction, t being the binary ‘treatment effect’ covariate. By applying a likelihood ratio test, it is assessed whether the FP functions are the same in each t group, which is a test of the interaction. The t by x interaction model has 3 df (1 power, 2 β’s) for FP1 functions and 6 df (2 powers, 4 β’s) for FP2 functions of x. The corresponding main effect models have 2 and 4 df respectively. The test of interaction, therefore, has 1 and 2 df for FP1 and FP2 functions, respectively.

The difference for the first variant (flex 2, Table A1) is that the FP powers are determined in a model assuming an interaction, constraining the powers of x to be the same for each level of the treatment variable. The same powers are then used to model the main effect of x. The flex2 approach may result in selecting powers different from those with flex1 and therefore in a different test outcome. The df are the same as for flex1. The second variant (flex3, Table A1) takes the same approach to the interaction as flex2, but allows the power terms for the main effect to differ from those for the interaction. The test of interaction assumes 1 and 2 df for FP1 and FP2 functions, respectively. The third variant (flex4) is the most flexible since it also allows the FP powers to differ at all levels of the treatment groups. The df for the tests of interaction are taken as 2 for FP1 and 4 for FP2 models.

Significance tests for interactions in the flex2, flex3 and flex4 variants are based on the chi-square distribution with the stated df. However, the tests are non-nested, and the resulting *p*-values lack theoretical underpinning. The significance levels may therefore be liberal or conservative. The four variants are available via the flex( ) option of the MFPI routine for Stata [8].

Based on results from a large simulation study, Royston and Sauerbrei chose an FP1 function with flex3 as their preferred approach (Table A1), provided there are no clinical arguments that TEF may be non-monotonic. In the latter case an FP2 function is needed [10]. Here we use FP1 (flex3). We also investigate FP2 (flex1) functions as a sensitivity analysis.

The MFPI approach supports adjustment for possible confounding variables. From a clinical point of view, progesterone receptor values and age would be obvious candidates. However, these variables were not available to us, therefore all analyses are unadjusted.

### Treatment effect function

Using the power terms determined for each treatment group, MFPI estimates an FP function representing the prognostic effect of the continuous covariate of interest, optionally adjusting for other covariates [5, 30]. The difference between the functions for the treatment groups is calculated and tested for significance. The testing is performed through an analysis of interaction between treatment and the FP function. A plot of the difference and related CI, for example odds ratio or hazard ratio (HR), against the covariate, gives a graphical representation of the resulting TEF. A TEF for a continuous covariate not interacting with treatment would be a straight line parallel to the x-axis, whereas a treatment-covariate interaction would be indicated by a non-constant line or curve, often increasing or decreasing monotonically. We refer to [5] for a description of the relationship between our interaction model and Hastie and Tibshirani’s varying coefficients models [31] and for formulas for FP based TEFs.

As proposed by Royston and Sauerbrei we check estimated TEFs by considering subgroups defined by increasing values of estrogen [5]. Here we use four subgroups with cutpoints determined by the 10,30, and 50 centiles of ER in the overall population. Groups are 0–5, 6–29, 30–76 and > 76 (see Table A2 for results). The chosen centiles put more emphasis on small ER values and we used these specific centiles in an earlier analysis for a similar patient group (see Table 7.5 in [6]).

### Meta-analysis of functions

When IPD for several studies are available, Sauerbrei and Royston proposed a new meta-analysis strategy to summarize the functional relationship between a continuous variable and the outcome through a regression model [12]. Here we use this two-stage approach to derive a summary treatment effect estimate from the individual functions in the eight randomized trials. The approach was designed for observational studies, usually involving confounder variables. The procedure comprises three steps. First, a confounder model is determined. This step is not needed here. Next, in each study the functional form for the continuous variable of interest is estimated, adjusted for the confounder model, if required. Finally, weighted averaging is used to combine the individual functions, aiming to obtain a summary estimate of the functional relationship.

In a meta-analysis, the weight attached to each study is usually determined by criteria such as the sample size or (for a survival outcome) the effective sample size, and (inverse) variances of estimates of interest. However, such criteria do not reflect different possible distributions of a continuous variable across studies. Depending (among other things) on inclusion and exclusion criteria, such distributions may vary considerably between the studies. A study may have little or no information in a specific range of the data and a correspondingly wide CI of the treatment effect function. To reflect this local paucity of information in the meta-analysis, Sauerbrei and Royston proposed pointwise averaging of the functions, the weights for each study depending on the information at each distinct covariate value [12]. This approach is very general and could be used to combine different types of TEFs (e.g. various types of spline function) in exactly the same way. In addition, fixed or random effects assumptions lead to different variance structures and correspondingly different weights. We show results for both fixed and random effects models.

For our meta-analysis approach of treatment effect functions the name metaTEF is used.

## Results

To be transparent and better to guide the reader through all analyses conducted, we provide in part C of the MethProf-MA profile (Table 1) an overview of all the steps of our analysis.

### Preliminary analyses

For each study we investigated whether an interaction of chemotherapy with estrogen receptors exist. *P*-values of the corresponding test (Table 2) show a highly significant effect in trial 8 but none of the other seven *p*-values are below 0.05. That is to be expected because none of the trials was powered to investigate for interactions. Estimating the effect of chemotherapy in various subgroups with changing ER values provides a crude assessment for a potential interaction. For the four groups considered, estimated hazard ratios for the effect of CT increase monotonically from 0.70 for ER up to 5 fmol (subgroup 1), 0.78 (subgroup 2), 0.89 (subgroup 3) to 0.93 for ER > 76 fmol (subgroup 4, Table A2). This indicated that the effect of chemotherapy is much larger for low ER values.

For each individual trial we show Kaplan-Meier estimates for the four subgroups (Fig. A3). Only 1 of the 16 subgroup analyses for larger ER values (30–76 and larger than 76) point to a relevant effect of chemotherapy (subgroup 3 in trial 8) whereas we see some large effects in subgroup 1 (trials 1,3,6).

In a pooled analysis of all data, the log function (power term 0) was selected as the FP1 function to best describe the relationship between ER values and the outcome. Figure A4 gives the corresponding eight functions from the single studies and the corresponding pooled estimate. Whereas the pooled estimate and the single estimates from six studies increase with ER values, two of the eight functions (studies 4 and 5) point to an effect in the other direction. The pooled estimate (TEF, log HR) increases from about − 0.4 (ER value 0) to about 0 (ER value 1000).

### metaTEF

**Fig. 2:**
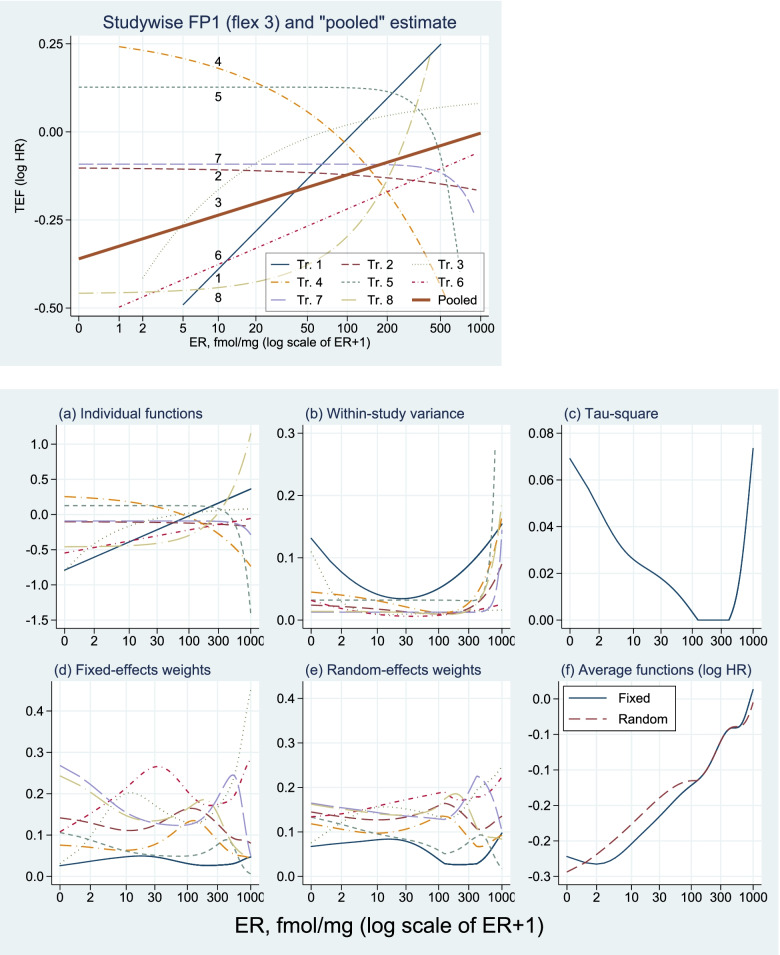
Studywise TEFs (FP1, flex3) (top with restricted TEF scale). Bottom part: the same TEFs with full scale (**a**) and corresponding variances, weights (random effects and fixed effects) and metaTEFs. For comparison the ‘pooled’ estimate is also given

Results of our main analysis with the studywise FP1 approach are shown in Fig. 2. Different power terms are selected for each study and the corresponding TEFs vary much more than in the pooled analysis shown in Fig. A4, but as before study 4 points to an effect in the ‘wrong directions’, the effect of CT increases with increasing ER values. The function for study 5 points to an unrealistic effect for very large values, probably caused by a small number of influential points. However, such extremes of some functions have also a very large variance (see (b) in the right part) and consequently the weight at extreme values is very low. Within study variance functions are shown in part (b) and between study variances in part (c). Specifically, for fixed effect weights, the ER distribution is reflected in the weights. The functions for the corresponding random effect weights are less wiggly, reflecting that differences between ER distributions are less relevant for these weight functions. The averaged treatment effects are shown in part (f) of Fig. 2. Whereas the fixed effect function has a minimum at 2 fmol (we modelled ER + 1, therefore the true ER value is 1; see “Estrogen receptor” and “General issues of the three-stage metaTEF approach”), the random effect function is strongly monotonic. Altogether, the two functions are very similar and clearly demonstrate that the hazard ratio increases with an increase in ER values.

### Sensitivity analyses

**Fig. 3:**
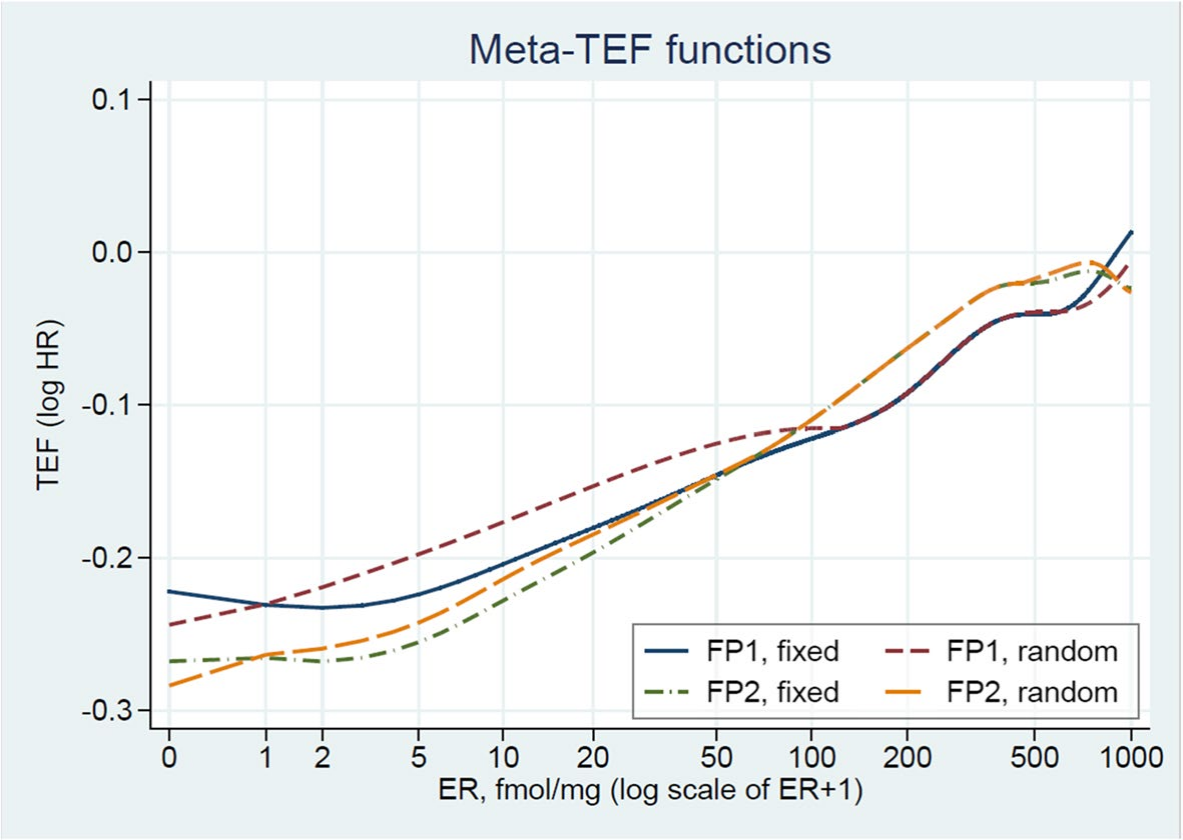
metaTEF functions for approaches FP1/FP2 and fixed and random effects

In Preliminary analyses we presented analyses in subgroups, which may be considered as preliminary analyses, a sensitivity analysis or a check of the analysis based on fractional polynomial modelling. An important sensitivity analysis is whether results differ if we allow more flexible FP2 functions. Studywise TEFs and the pooled estimate are given in Fig. A5, *p*-values for the test for interaction are given in Table A3. The FP2 class allows more flexible functions which results in larger variability between the functional forms. Apart from a small difference for ER around 700 and above, the averaged functions are similar. For all four combinations of FP1/FP2 and fixed/random effect models the metaTEFs clearly show a strong influence of ER on the effect of chemotherapy. The only small clinically relevant difference can be seen for very low values (eg 2 or 3 fmol, fixed effect models differ from random effect models) and for large values (around 800 fmol) where FP2 models may point to a cutpoint (Fig. 3). However, variances are large in these regions (Fig. A6).

## Discussion

In the following we start by discussing our findings from the breast cancer example. We continue by discussing general methodological issues of our metaTEF procedure, mention alternatives and compare results with the meta-STEPP approach. We stress the importance of complete reporting with a structured profile and discuss strengths and limitations of our approach.

### Results of metaTEF for the DataMart studies

For early breast cancer patients treated with tamoxifen, the metaTEF functions provide convincing evidence of an interaction between chemotherapy treatment and estrogen receptor values. Whereas CT has hardly any effect for larger (say > 500 fmol) values, the log hazard ratio is monotonically increasing from about − 0.25 for ER ‘0’ to about 0. An overall test for an interaction is significant (*p* = 0.0215) but the estimated treatment effect function is a much stronger argument for the interaction. As individual RCTs are typically underpowered for exploring patient characteristics interacting with treatment [32] it is no surprise that only one of the eight studies (study 8) pointed towards a significant interaction. Three of the studies (4, 7, 8, see Fig. A2) included very few patients with ER below 10 fmol, a potential reason that two of the corresponding individual treatment effect functions had a negative slope (Fig. 2). This IPD meta-analysis clearly shows that such approaches are needed to provide evidence of whether individual studies are too small. Effective sample size (number of events) ranged from 127 to 712 in the eight studies, too low to investigate a treatment covariate interaction in single studies. Irrespective of using a fixed or a random effects model and whether an FP1 or an FP2 function is chosen, the main finding from the metaTEF approach provides clear evidence of an interaction between ER and CT. FP2 functions point to slightly larger effects for low values and the fixed effect models are flat for ER values up to about 5 fmol, whereas the random effect functions increase even in this area.

### General issues of the three-stage metaTEF approach

To investigate for an interaction between a continuous predictor and treatment, metaTEF combines three stages. First, the derivation of the functional relationship with fractional polynomials in both treatment groups (extension for more than two groups is straightforward); second, the estimation of a continuous TEF as the difference between the functions in the two groups; and third, the averaging of the TEF from each study. The first stage requires deciding between an FP1 or an FP2 function (FP3 or FP4 are possible but unlikely to be needed) and a decision between several variants. A simulation study [10] provided arguments for FP1 (flex 3) as the preferred option. FP2 (flex1) is the preferred approach if non-monotonic functions are expected [10]. See [13] for an example. It is advisable to use one of the approaches for the main analysis and the other for a sensitivity analysis.

TEFs from single studies show considerable variation and using a monotonic FP1 function within each study does not logically imply that the overall TEF will be monotonic. In our case study, the TEFs seem to suggest that in some studies there is no effect of ER values on the effect of CT or even that the effect points in the ‘wrong’ direction. However, with small sample sizes in many of the studies, that is not surprising. To avoid difficulties caused by too-small studies, Royston and Sauerbrei [9, 10] used sample sizes of 250 and 500 in their simulation study with a continuous outcome. Since it is also known that single points can have a major influence on FP functions selected, we decided to truncate ER values at 1000 fmol/l.

As the estimation of a treatment effect function with fractional polynomial methodology requires positive values for ER we used ER + 1 in the study. An extended FP approach was developed for variables with a spike at zero [33]. To our knowledge MFPI has not been extended to cover this situation. In principle it should be straightforward, and we would expect results to be similar to the simpler ‘standard’ approach used here.

### Potential alternatives for each of the three stages

FP functions in the first stage may be replaced by STEPP functions [14] (see below) or spline functions. In principle, splines are a natural alternative, but it is unclear which specific spline approach should be chosen. Regression splines with 2, 3 or 4 d.f. and automatic knot selection were considered in the simulation study mentioned above. Riley et al. show details of a meta-analysis with restricted cubic splines, but many more spline approaches are available [34]. Perperoglou et al. provide an overview of the most widely used spline-based techniques and their implementation in [35]. There is no ‘best’ spline approach. Further guidance is needed before a comparison of spline techniques with FPs will be able to provide important information for the selection of the most suitable approach to estimate a continuous TEF.

In the second stage, STEPP compares treatment effects (e.g. estimate of survival rate at 5 years or hazard ratios) in subgroups. This means that treatment differences are calculated for patients belonging to k (overlapping) intervals. If the pointwise approach is used in the third stage, it is possible to use any approach which estimates a functional relationship in each of the treatment groups and estimates the treatment difference with related variance for each point in a relevant interval.

In the third stage we use the two-step approach with pointwise weighted averaging of the derived study specific TEFs. Weights depends on the variances of the individual functions and therefore on distributions of the predictor in each study. Differences between studies are also used in the random effects approach, which implies that differences of predictor distribution are down weighted. In the context of the assessment of risk factors, White et al. [19] compared the pointwise approach with a multivariate meta-analysis procedure (‘mvmeta’) which combines the set of regression coefficients from each study [36–38]. In the latter, study specific estimates based on the same type of function are required. Linear functions per study are simplest but non-linear functions are possible if common powers are used across studies. Under these restrictions it would be possible to describe the individual TEFs with a set of regression coefficients and a multivariate meta-analysis would be possible. Results from the two approaches showed only minor differences in a very large IPD meta-analysis of risk factors (> 80 cohorts) but the pointwise approach is more flexible [19].

### Comparison with meta-STEPP

To extract all information from a continuous variable, Bonetti and Gelber [14, 15] proposed the ‘Subpopulation Treatment Effect Pattern Plot’ (STEPP), a graphical tool to elucidate the pattern of treatment-covariate interactions in two-arm clinical trials with time-to-event endpoints when the covariate of interest is continuous. The primary advantage of STEPP is that it is very intuitive –no functional form for the interaction needs to be specified, the method is based on the use of traditional measures of treatment effect on well-defined, overlapping subgroups of patients, and that it allows one to explore the pattern of possible treatment effect heterogeneity [18]. Subgroups can be defined in two different ways, known as sliding window (SW) and tail-oriented (TO). Differences of estimates in subgroups are arguments for an interaction of the prognostic factor with treatment. Related significance tests were proposed [39].

However, STEPP has disadvantages as a tool for inference and estimation. There are the two variants (SW and TO) to choose between. The size of the subpopulations is critical to the performance of the method and hence to the interpretation of the results. That is a specific issue for the SW variant, as shown for a single study by Sauerbrei et al. [17]. For a fixed effects meta-STEPP analysis Wang et al. [18] propose to create ER subpopulations based on meta-windows which use the data from the joint distribution of all studies. Consequently, some studies can have small sample sizes and some of the individual functions fluctuate substantially. A random effects meta-STEPP approach was proposed in [40]. There are only small differences between the fixed and random effect approaches. In agreement with the FP results, the plots show a clear increasing trend in the treatment effect as ER value increases, suggesting that the magnitude of the chemotherapy effect is smaller for tumours with higher levels of ER.

While metaTEF allows additional variables (prognostic factors, confounders) in a regression model, meta-STEPP cannot accommodate such variables. However, the issue is not critical here since we use data from eight randomized trials of chemotherapy with no covariates other than ER.

### Good reporting to help assessment of credibility

In Table 1 we introduce the three-part profile MethProf-MA as an instrument to improve reporting of available data and of all steps in the analysis. With an emphasis on the latter, Altman et al. [23] proposed the REMARK profile, a structured display in the context of prognostic factor research. Created prospectively, the profile helps by pointing out relevant issues, such as the necessity of initial data analysis and checking of important assumptions of models used [41]. Concentrating on all steps of our analysis, we adapt the key ideas of the REMARK profile to methodological investigations, here to a meta-analysis. Obviously, such profiles can also be used to better present and understand investigation of properties and comparison of variable selection procedures, simulations studies and many more [42] and call it a MethProf-MA profile, relating it to the reporting guidelines for meta-analyses. In our methodological presentation we use the data from an earlier meta-analysis which means that the PRISMA statement [24] is less relevant here. A key feature is the illustration of all steps of the analysis conducted, which can be easily seen in part C of MethProf-MA. The profile will also help to assess the credibility of effect modification analyses with ICEMAN (Instrument to assess the Credibility of Effect Modification Analyses), an instrument recently developed for randomized trials and meta-analyses [43]. The version for RCTs includes 5 core questions and that for meta-analyses 8 core questions, 4 of which overlap. One of the overlapping core questions is ‘If the effect modifier is a continuous variable, were arbitrary cutpoints avoided?’. Clearly this emphasizes the use of the full information from a continuous variable, as done with MFPI and the related metaTEF approach. A recent systematic survey clearly showed the necessity of assessing whether claims of subgroup effects are supported by the available data [44]. Other researchers may use ICEMAN to assess the credibility of the investigated interaction between estrogen receptor values and chemotherapy in patients with early breast cancer treated with tamoxifen. The overview provided by the profile will be most helpful for assessing some of the criteria.

### Strengths and limitations

MetaTEF is an approach which uses the full information from a continuous variable to assess whether and how effect of a treatment is modified by the variable. It avoids the use of cutpoints with their related critical issues [4]. It extends the MFPI approach, which has sufficient flexibility to model non-linear treatment effect functions in many cases. MFPI has greater power than several alternatives [10] and provides functions which are simple, understandable and transferable. Related metaTEF functions are pointwise weighted averages of FP functions and are therefore complex. The resulting functional form cannot be described by a simple formula. Smoothing the pointwise average and variance functions may help to increase visualisation and practical use but it is beyond the scope of the paper.

Provided IPD data is available from all relevant randomized trials, metaTEF summarizes all relevant information concerning a treatment modifying effect of a continuous variable and can even include further prognostic factors. This may increase the power of an analysis and may point to other factors which may modify the treatment effect. Age and progesterone receptor would be of interest in our example, but these data were not available to us. At a first glance MFPI modelling is straightforward, but there is the danger that some of the studies may be (too) small. In such cases it is likely that the algorithm will select a linear function in each group, even though the true effect may be far from linear. Mismodelling is also an issue if outliers or influential points are present. To cope with such issues, we suggest selecting a suitable truncation point. Also, we note that data-dependent modelling introduces bias in the estimates of the regression coefficients and that variances are underestimated [13].

We used data which were previously analyzed by Wang et al. [18]. This avoided the considerable task of assembling and cleaning the data, a complex and difficult issue in IPD meta-analysis. In addition, we have a clinically relevant and methodologically well-defined data set. It is unlikely that considerations such as publication bias are particularly relevant in our example. Furthermore, before starting the analysis there was no doubt that ER has an effect on chemotherapy. The main clinical questions relate to selection of a cutpoint for clinical decision making. In general, the treatment effect function will facilitate this decision. In our example, however, the TEF provides no clear answer.

## Conclusions

The demand to manage patients with individualized treatment strategies has increased considerably. Methodologically, however, individualized treatment plans imply that either a reliable prognostic classification scheme with heterogeneous treatment effects or treatment-covariate interactions exist and have been clearly identified.

Despite serious loss of power and other well-known critical issues, continuous variables are usually categorized or even dichotomized in regression models. With single trials, there are convincing arguments that the MFPI procedure has substantial advantages. Treatment effect functions are a simple way to illustrate effects in each study graphically. We show how to average them by applying a pointwise meta-analysis strategy. The metaTEF approach is transparent and allows TEFs to be combined, even if the distribution of the potential predictor differs between the studies (e.g. because of differences in the patient inclusion and exclusion criteria). Our suggested MethProf-MA profile provides an instrument for improving the reporting of meta-analyses.

## Supplementary Information

**Additional file 1.**

## Data Availability

The data may be available from the Breast Cancer DataMart Consortium. The Stata program metatef is available on the Statistical Software Components (SSC) archive. In the appendix we attach an annotated Stata do-file that creates Fig. 3.

## Abbreviations

BCDM: Breast cancer DataMart
CT: Chemotherapy
d.f.: Degrees of freedom
DFS: Disease free survival
ER: Estrogen receptor
flex1–4: Flexibility of a MFPI function (see Table A1)
FP: Fractional polynomial
FP1/FP2: Fractional polynomial of degree 1 or 2
HR: Hazard ratio
ICEMAN: Instrument to assess the credibility of effect modification analyses
IPD: Individual patient data
MethProf-MA: Profile for structured reporting (Meth: methodological, Prof: profile, MA: meta-analysis; see Table 1)
metaTEF: meta-analysis of treatment effect functions
MFP: Multivariable fractional polynomial
MFPI: Multivariable fractional polynomial interaction
Mvmeta: Multivariate meta-analysis procedure
PRISMA: Preferred reporting items for systematic reviews and meta-analyses
PROGRESS: Prognosis research strategy
RCT: Randomised controlled trial
REMARK: Reporting recommendations for tumour marker prognostic studies
RFS: Recurrence free survival
STEPP: Subpopulation treatment effect pattern plot
SW/TO: Sliding window/Tail-oriented, version of STEPP
TEF: Treatment effect function
