# Supplementary material for: Investigating treatment-effect modification by a continuous covariate in IPD meta-analysis: an approach using fractional polynomials
Source: BMC Med Res Methodol. 2022 Apr 6;22:98. doi: 10.1186/s12874-022-01516-w (PMC8985287; doi:10.1186/s12874-022-01516-w)

**Supplementary Material**

**More detailed results and a Stata script as an example for an analysis**

Tab A1

| Variant | Description |  | d.f. | |
| --- | --- | --- | --- | --- |
| Flex |  | | FP 1 | FP 2 |
| 1 | FP powers determined for main eﬀect of x;  use these same powers at each level of | | 1 | 2 |
| 2 | FP optimized over levels of t; use  these same powers at each level of t | | 1 | 2 |
| 3 | As flex2, but re-estimate powers  for main eﬀect of x | | 1 | 2 |
| 4 | Optimize FP for main eﬀect and  separately at each level of t | | 2 | 4 |

Variants of MFPI with various constraints, giving differing flexibility for the functions. d.f. stands for degrees of freedom for the tests of interaction. See the web appendix from Royston and Sauerbrei (2013) for details.

Tab A2

| ER | HR | SE | P | 95% CI |
| --- | --- | --- | --- | --- |
| 0-5 | 0.700 | 0.077 | 0.0012 | 0.564, 0.869 |
| 6-29 | 0.779 | 0.065 | 0.0027 | 0.661, 0.917 |
| 30-76 | 0.885 | 0.076 | 0.1561 | 0.747, 1.048 |
| >76 | 0.927 | 0.050 | 0.1586 | 0.834, 1.030 |

Preliminary analysis of pooled data. Estimated effect of CT (hazard ratio) in a Cox model for each of four ER subgroups, stratified by study.

Table A3

|  |  | FP1 | | FP2 | |
| --- | --- | --- | --- | --- | --- |
|  | trial | powers | p-values | powers | p-values |
| 1 | IBCSG-3 | 0 | 0.0917 | (-2,-2) | 0.2511 |
| 2 | IBCSG-7 | 0.5 | 0.8729 | (-0.5,-0.5) | 0.9963 |
| 3 | IBCSG-9 | -0.5 | 0.1198 | (-0.5,0) | 0.0390 |
| 4 | NCIC-MA4 | 0.5 | 0.2195 | (0,3) | 0.0289 |
| 5 | NSABP-B16-1 | 3 | 0.2444 | (3,3) | 0.6791 |
| 6 | NSABP-B16-2 | 0 | 0.1027 | (0.5,0.5) | 0.1585 |
| 7 | NSABP-B20 | 3 | 0.5155 | (3,3) | 0.3277 |
| 8 | SWOG-S8814 | 1 | 0.0081 | (1,1) | 0.0687 |
|  | Pooled | 0 | 0.0215 | (0,2) | 0.0136 |

Sensitivity analyses for the choice of the FP functions in the individual trials and for the meta-analysis (pooled data, stratified by trial). Comparison of (FP1, flex3; power 0 chosen) with (FP2, flex1; powers (0,2) chosen). p-values from the test for interactions.

Table A4


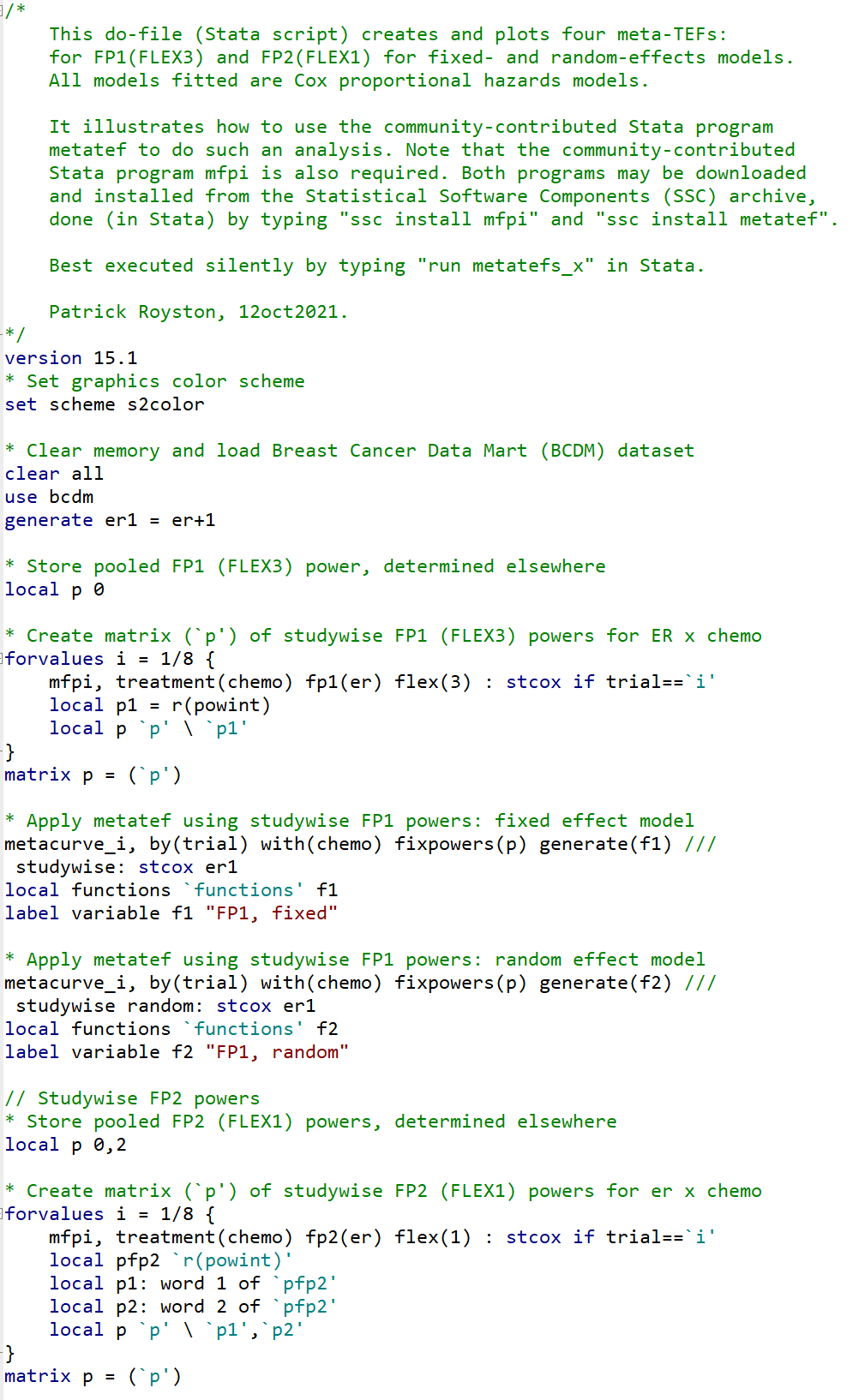


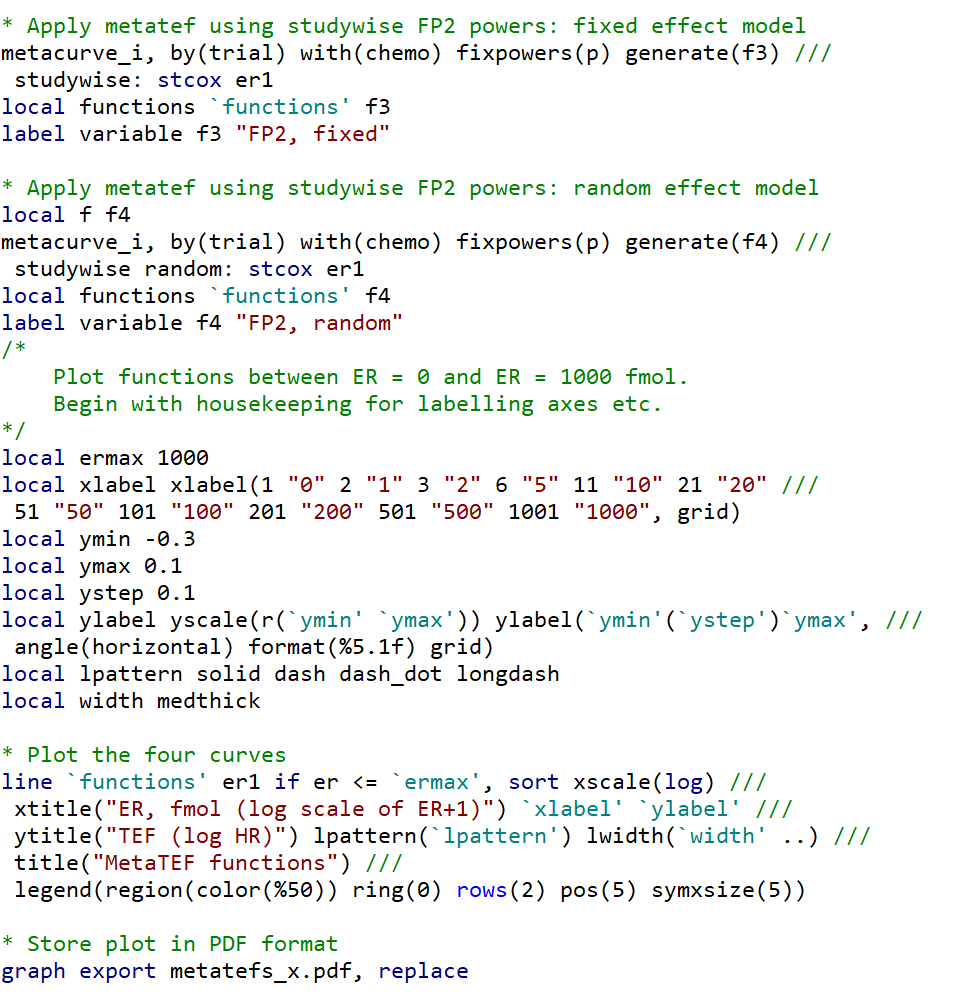


Stata script (called *metatefs_x.do*) to create and plot four metaTEFs for FP1(flex3) and FP2(flex1) for fixed- and random-effects models. All models fitted are Cox proportional hazards models.

Appendix figures

Figure A1: Effect of chemotherapy in each study. Please note the different lengths of follow-up times.


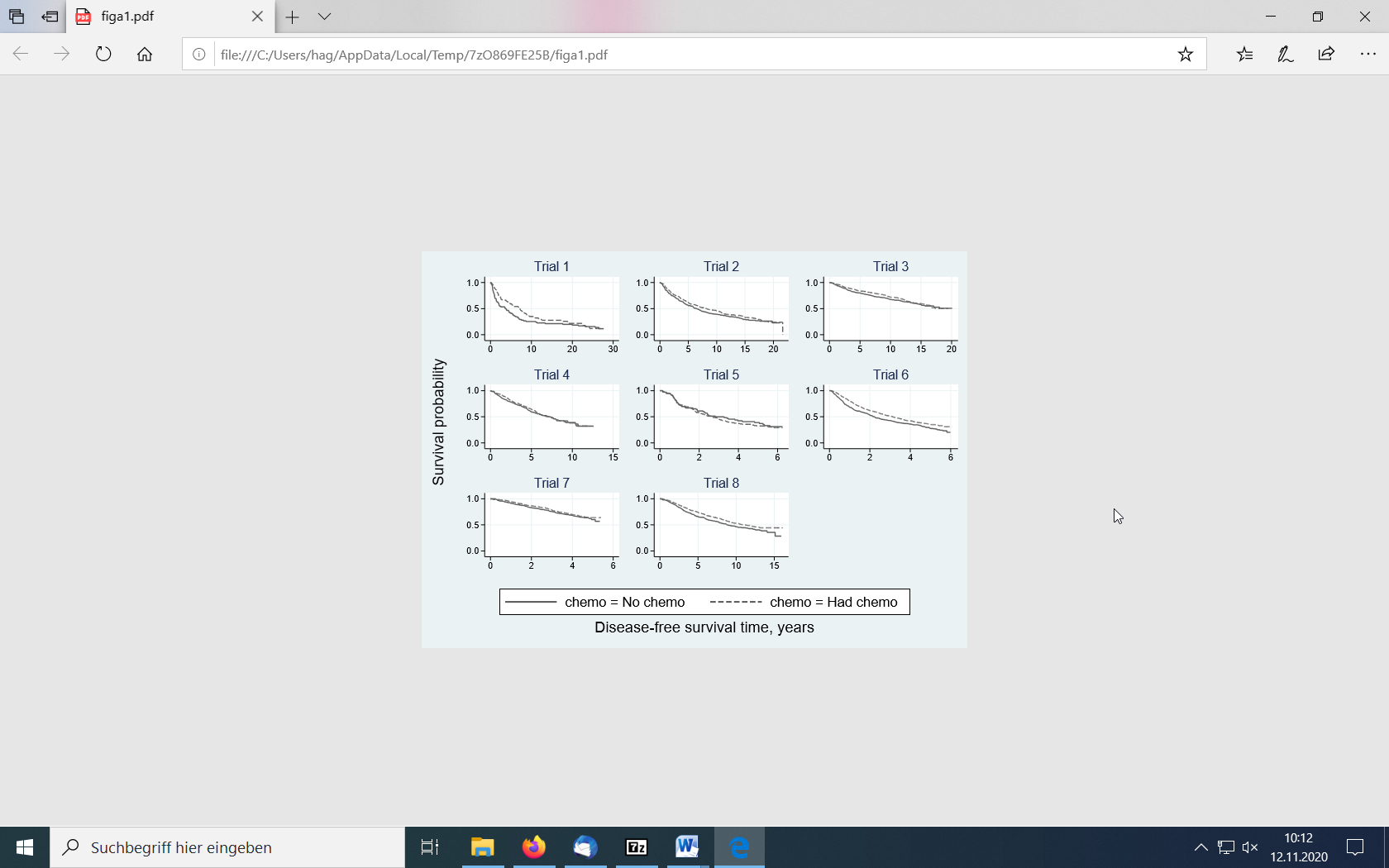


Figure A2: Distribution of ER per study
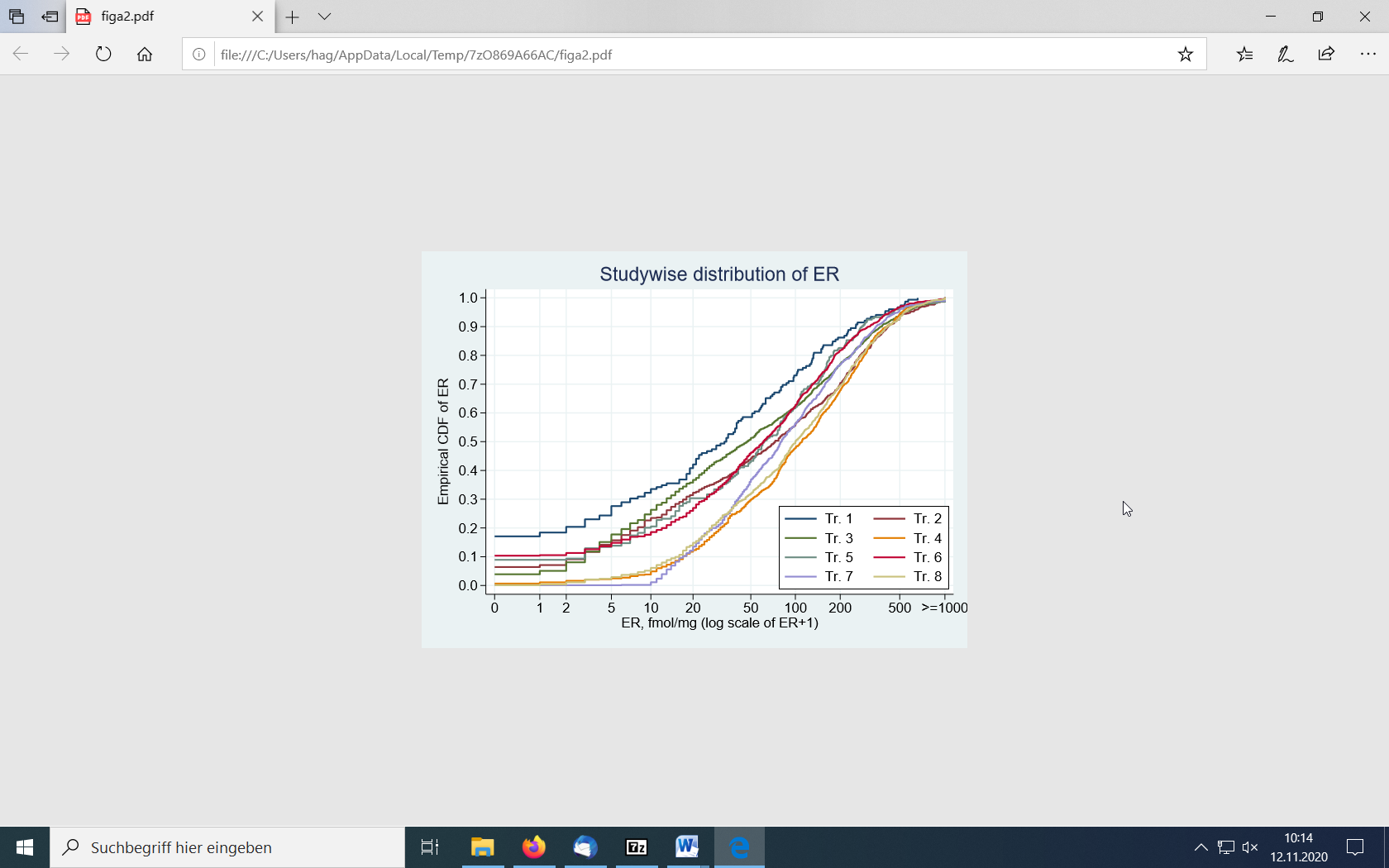


Figure A3: Preliminary analysis of single studies. Estimates of DFS in each of four ER subgroups. Please note the different lengths of follow-up times.


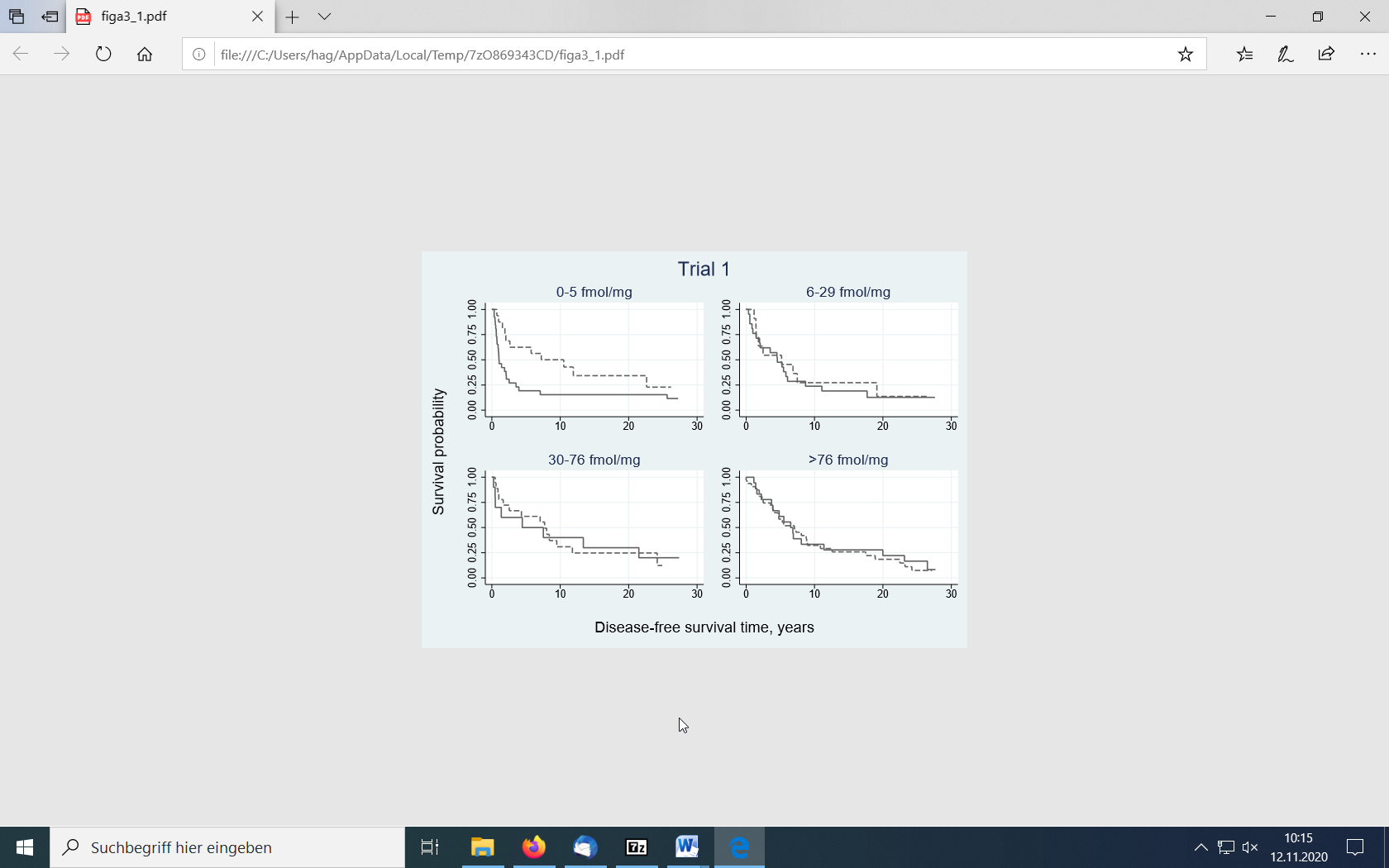

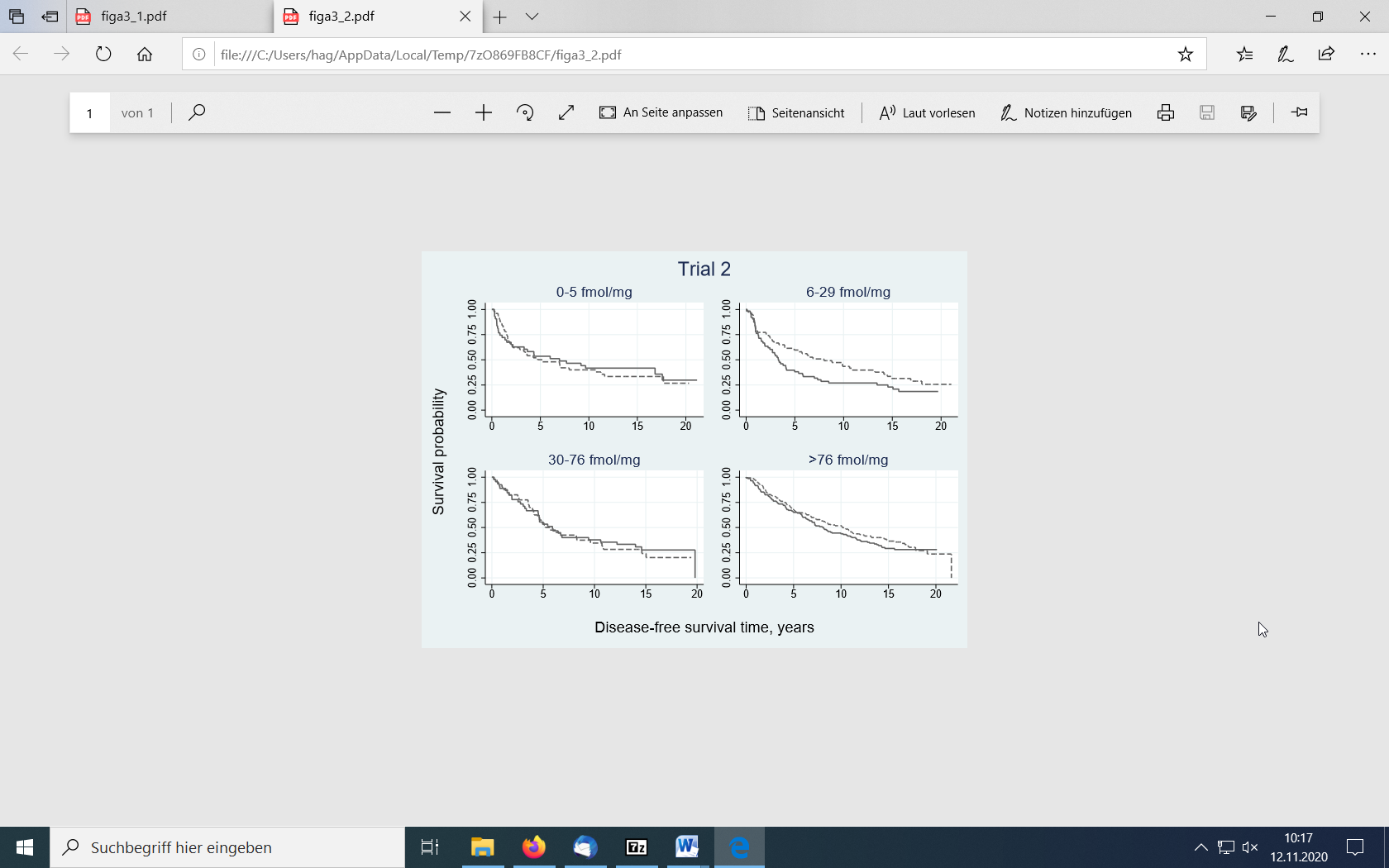


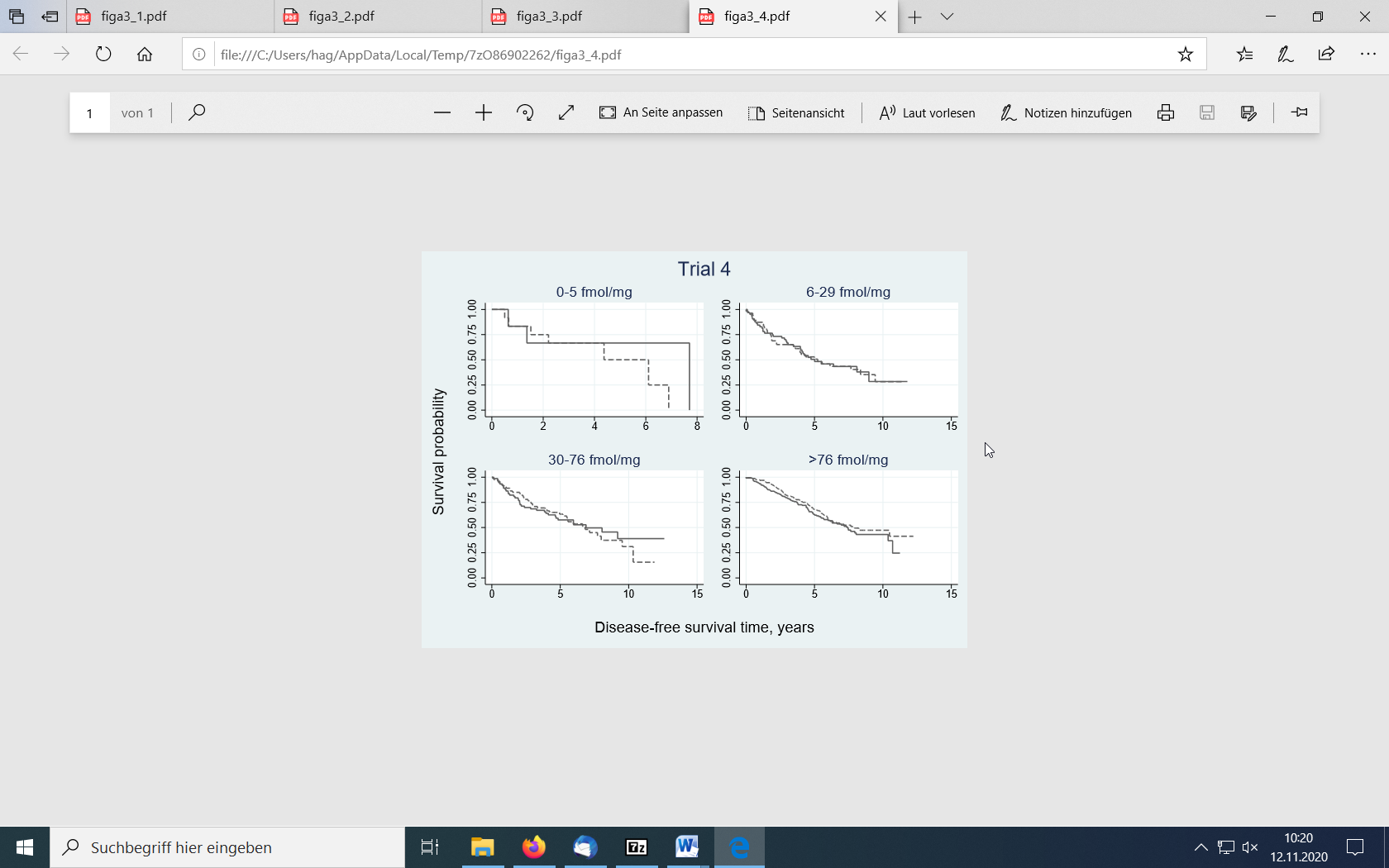

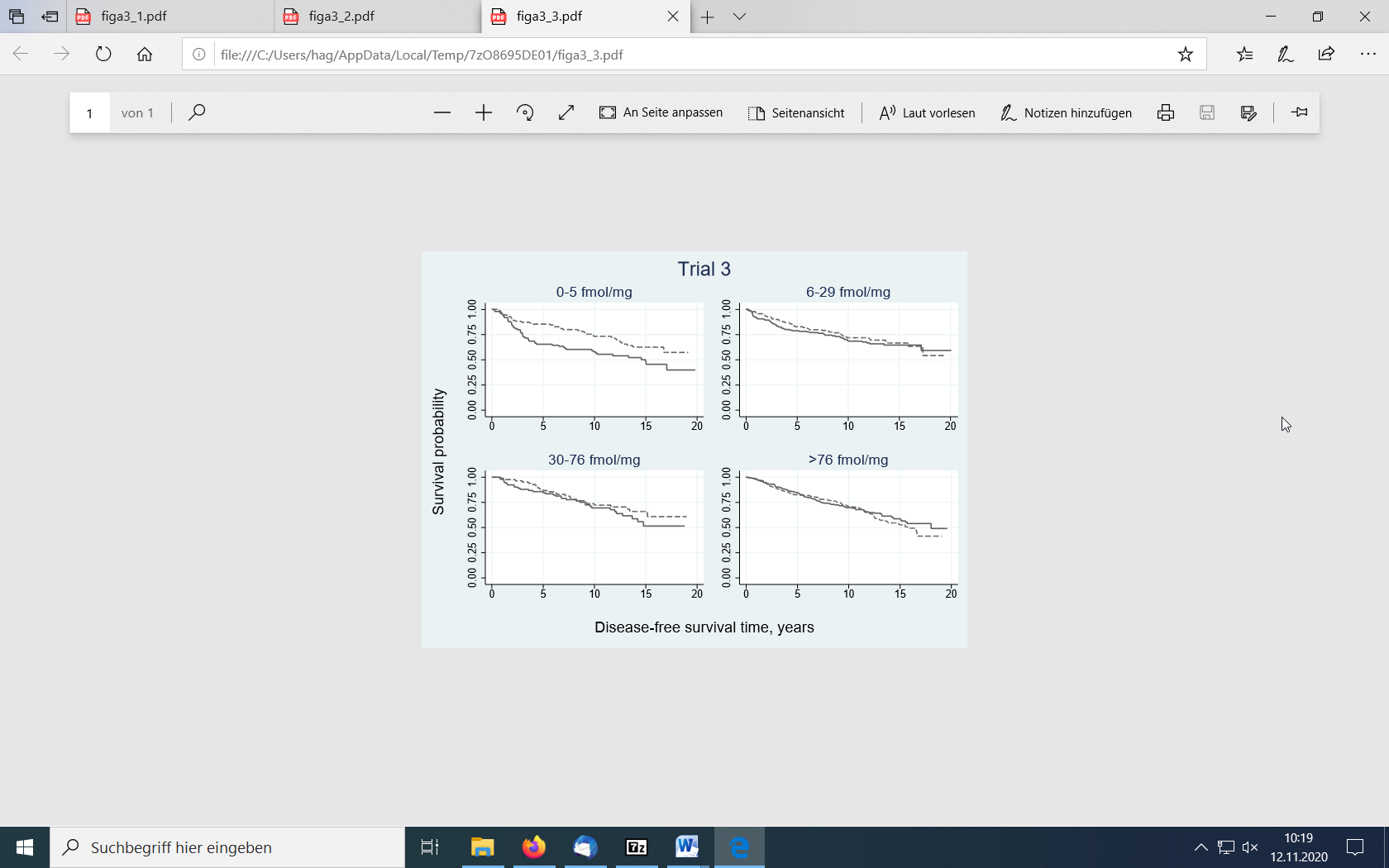


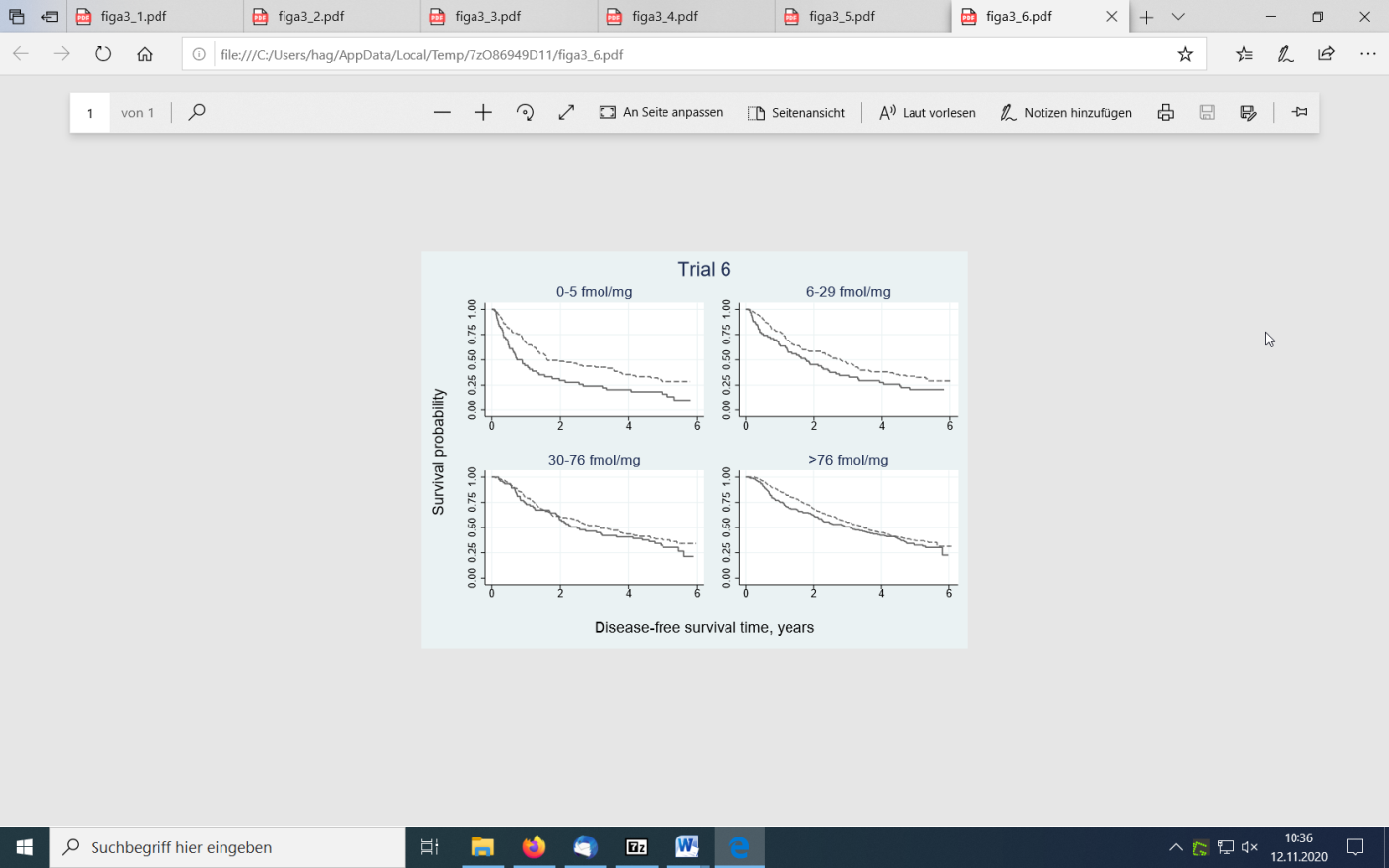

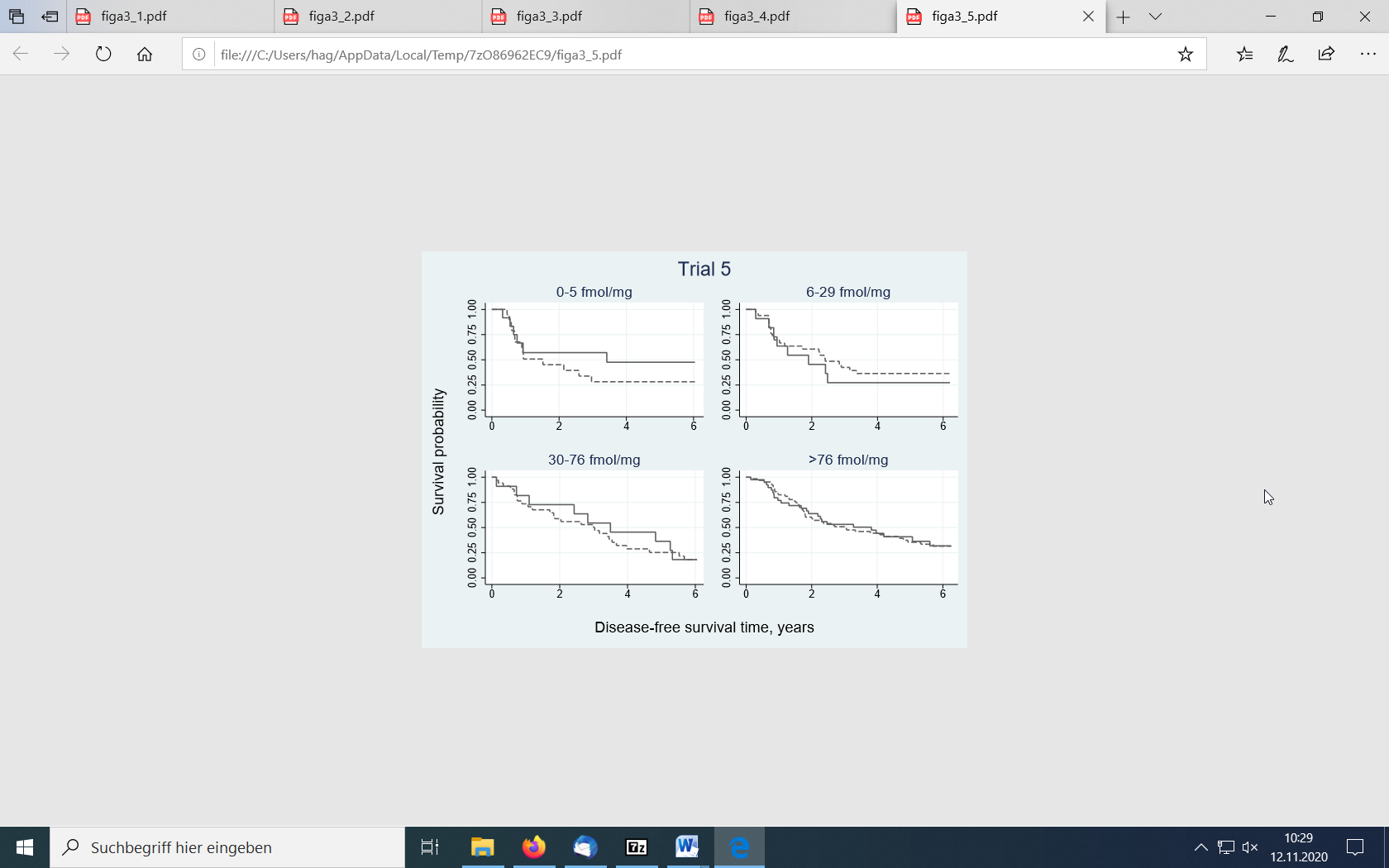


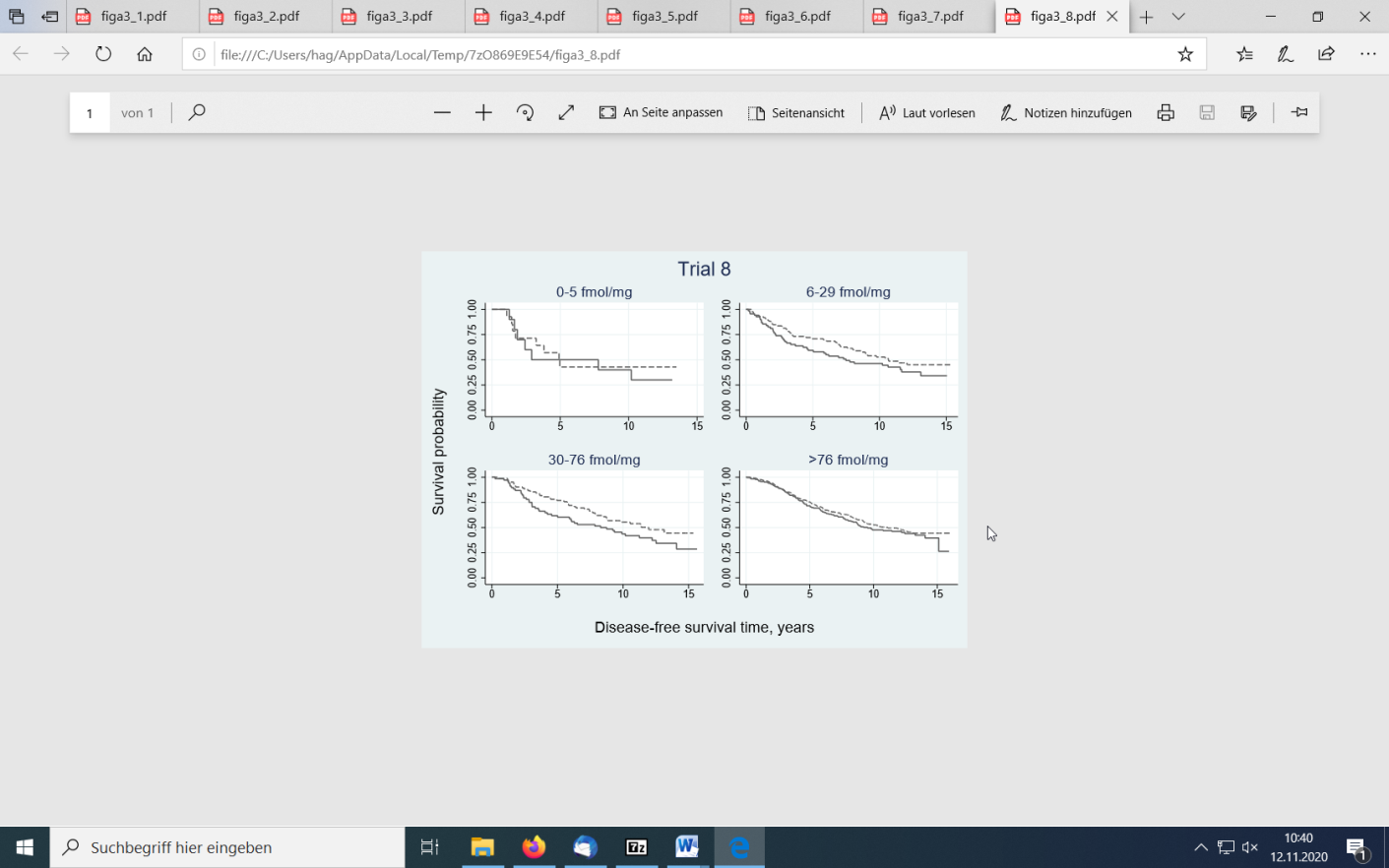

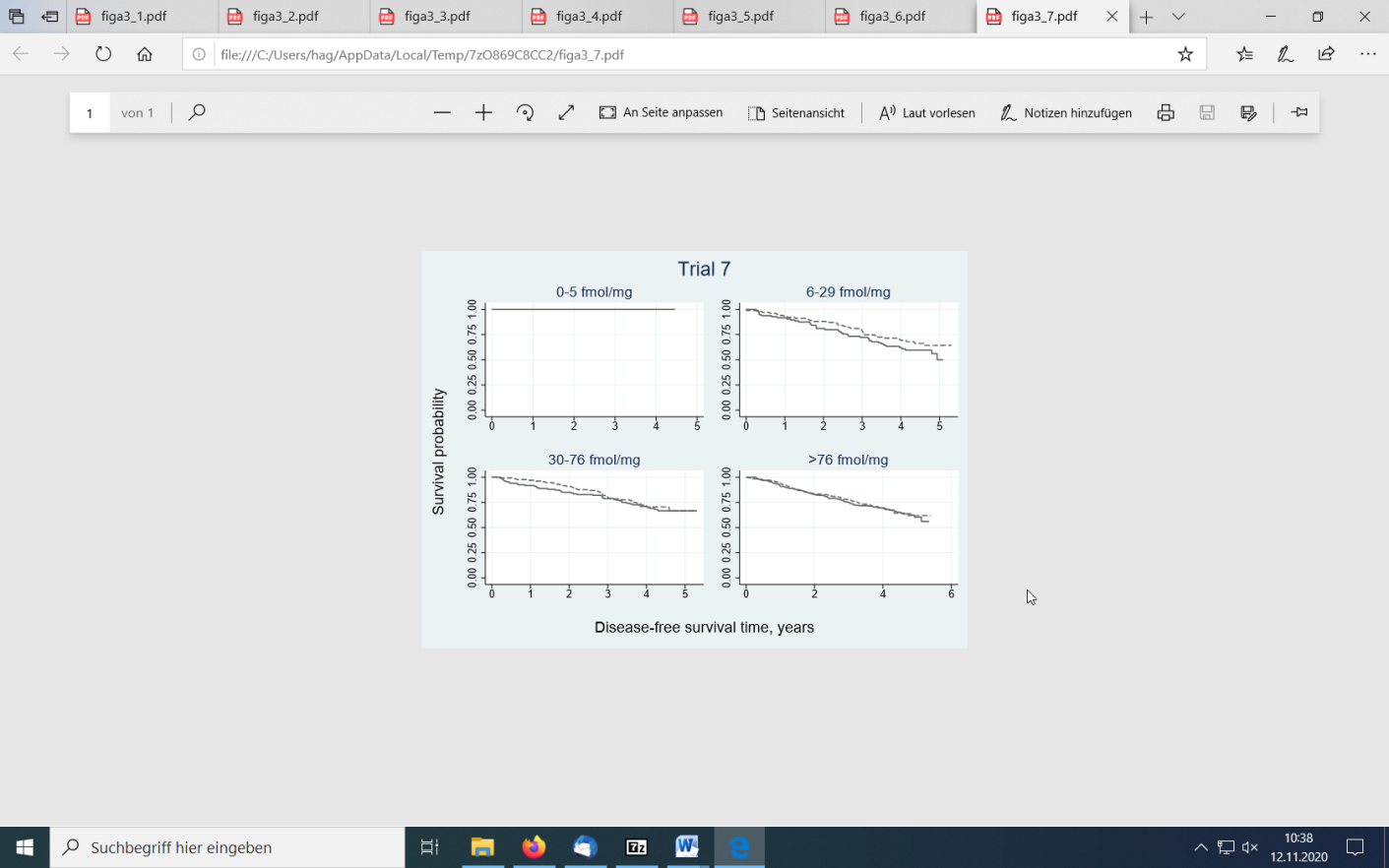


Figure A4: In the pooled analysis log(ER+1) was chosen as the best fitting FP1 function (correct). TEFs from the eight single studies and the pooled analysis. Please note the log scale of ER+1.


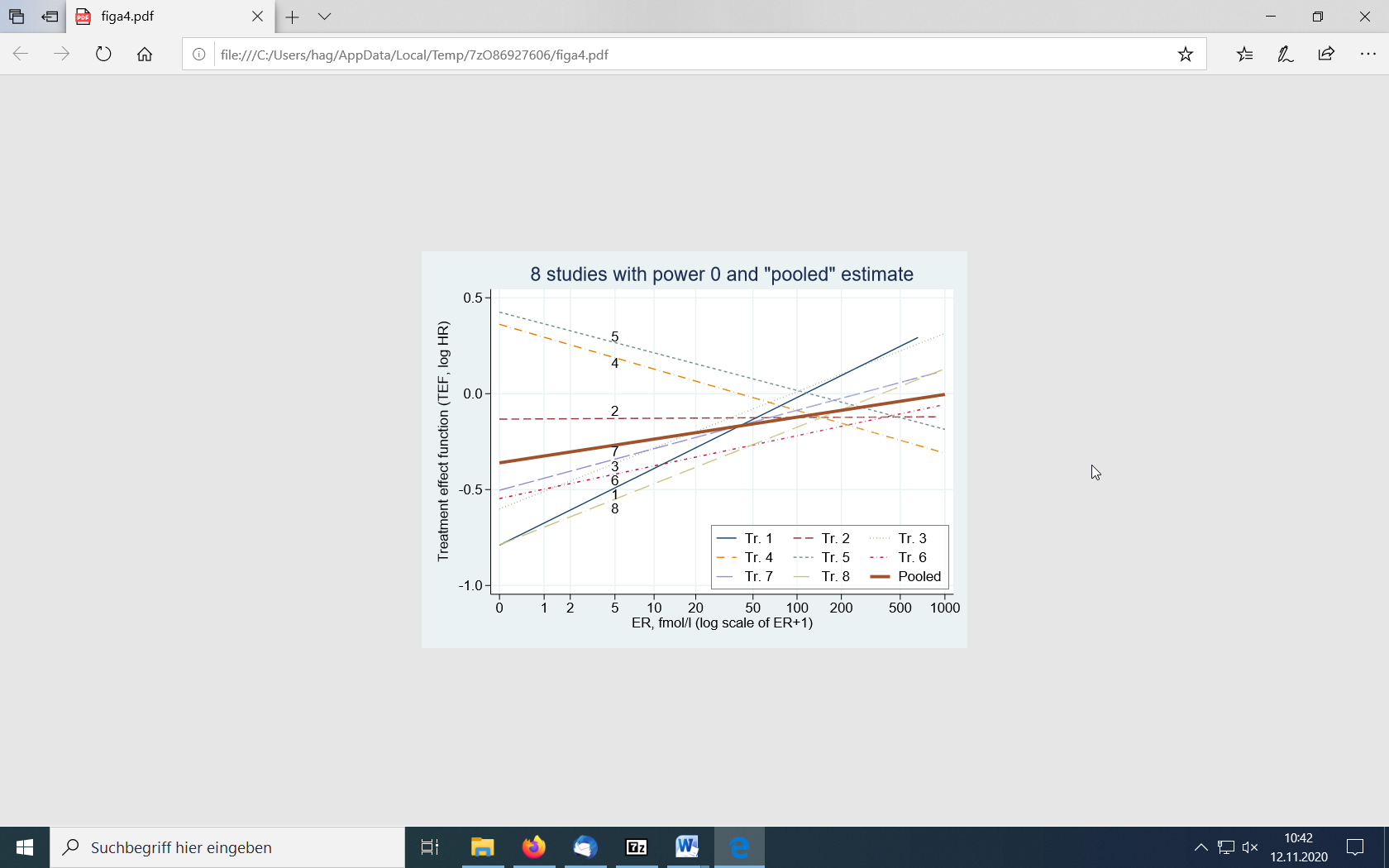


Figure A5: Studywise TEFs (FP2, flex1); for comparison the ‘pooled’ estimate is also given.


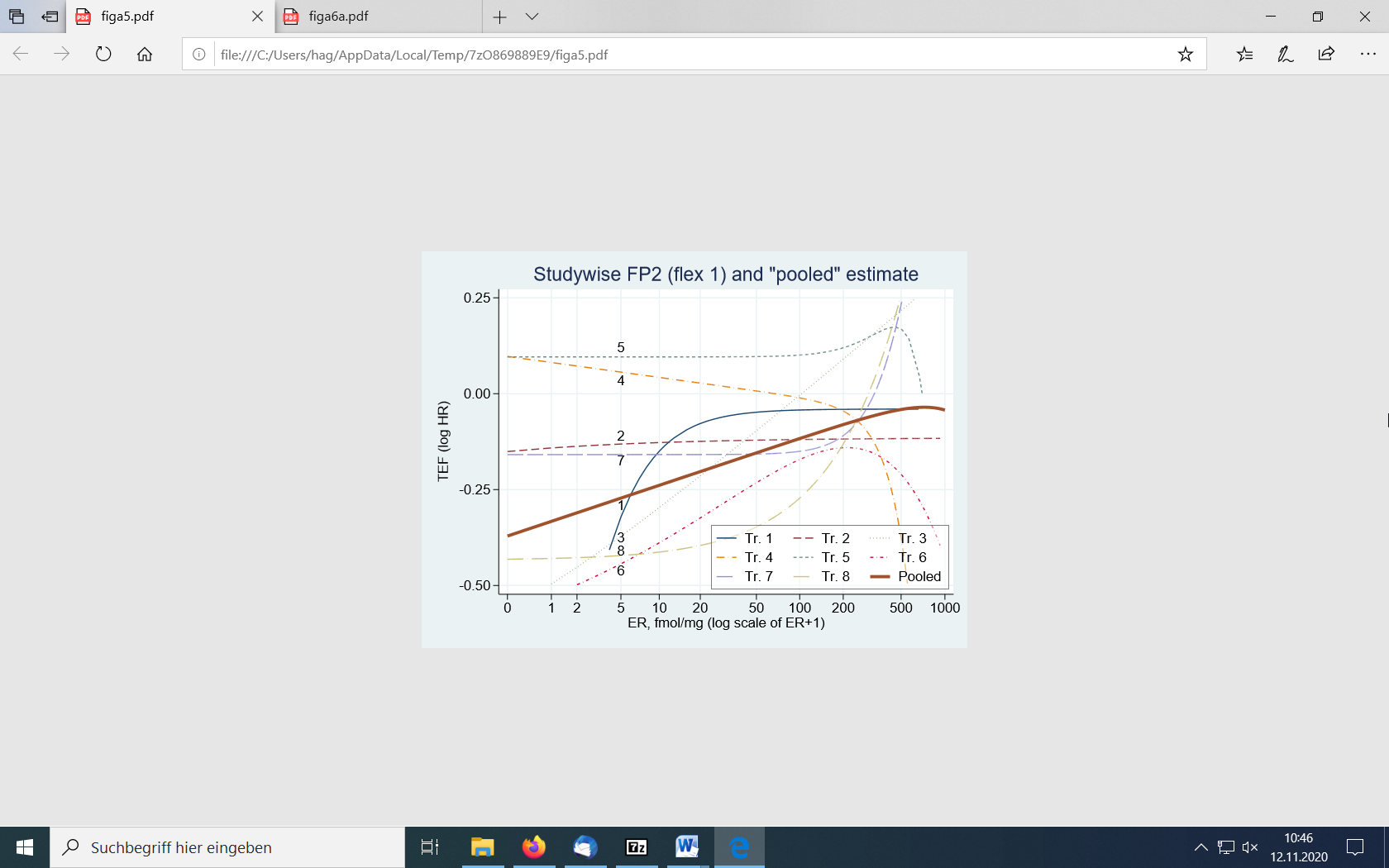


Figure A6a, A6b: Treatment effect function for fixed and random effect models, shadowed areas are 95% pointwise confidence intervals.


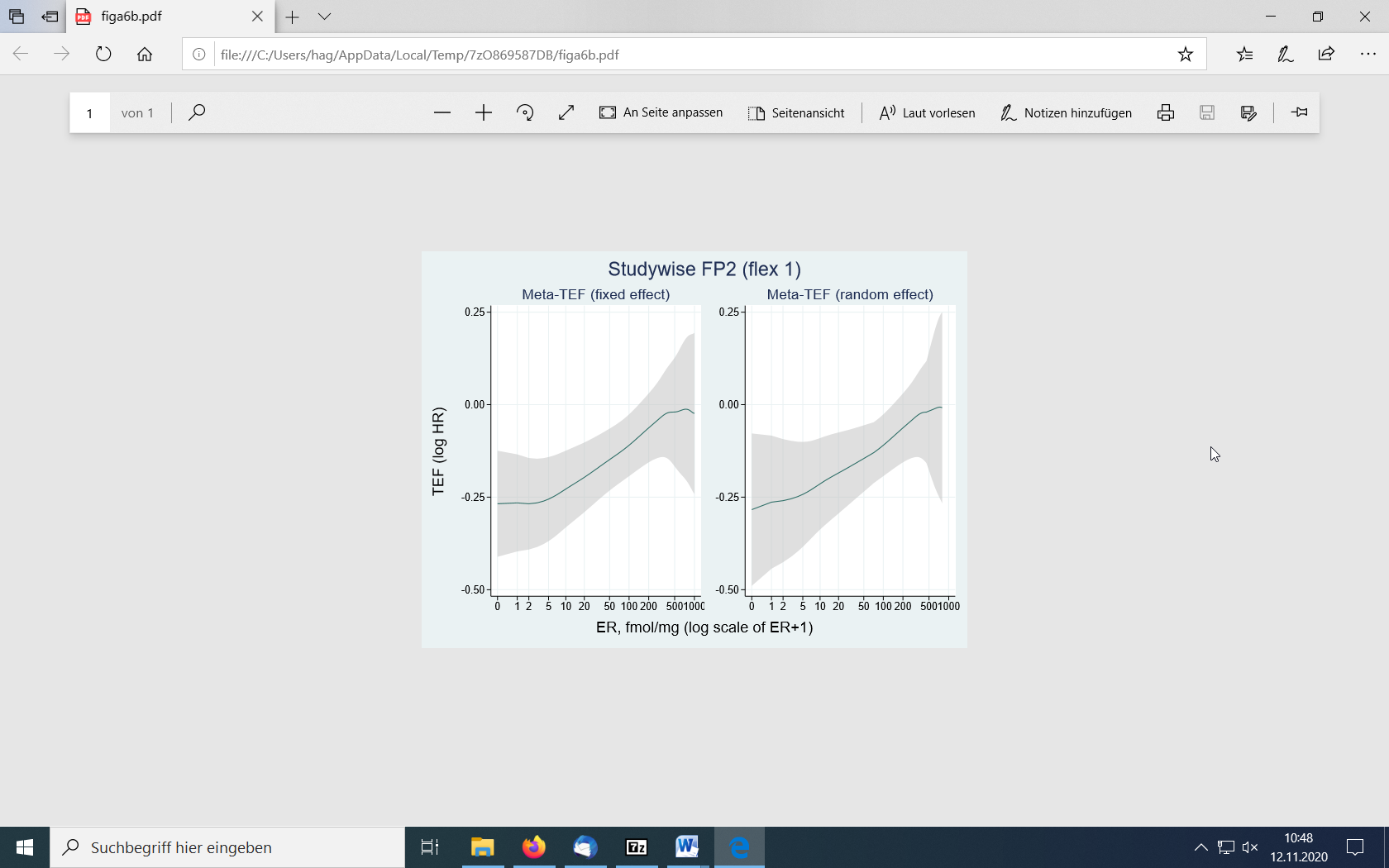


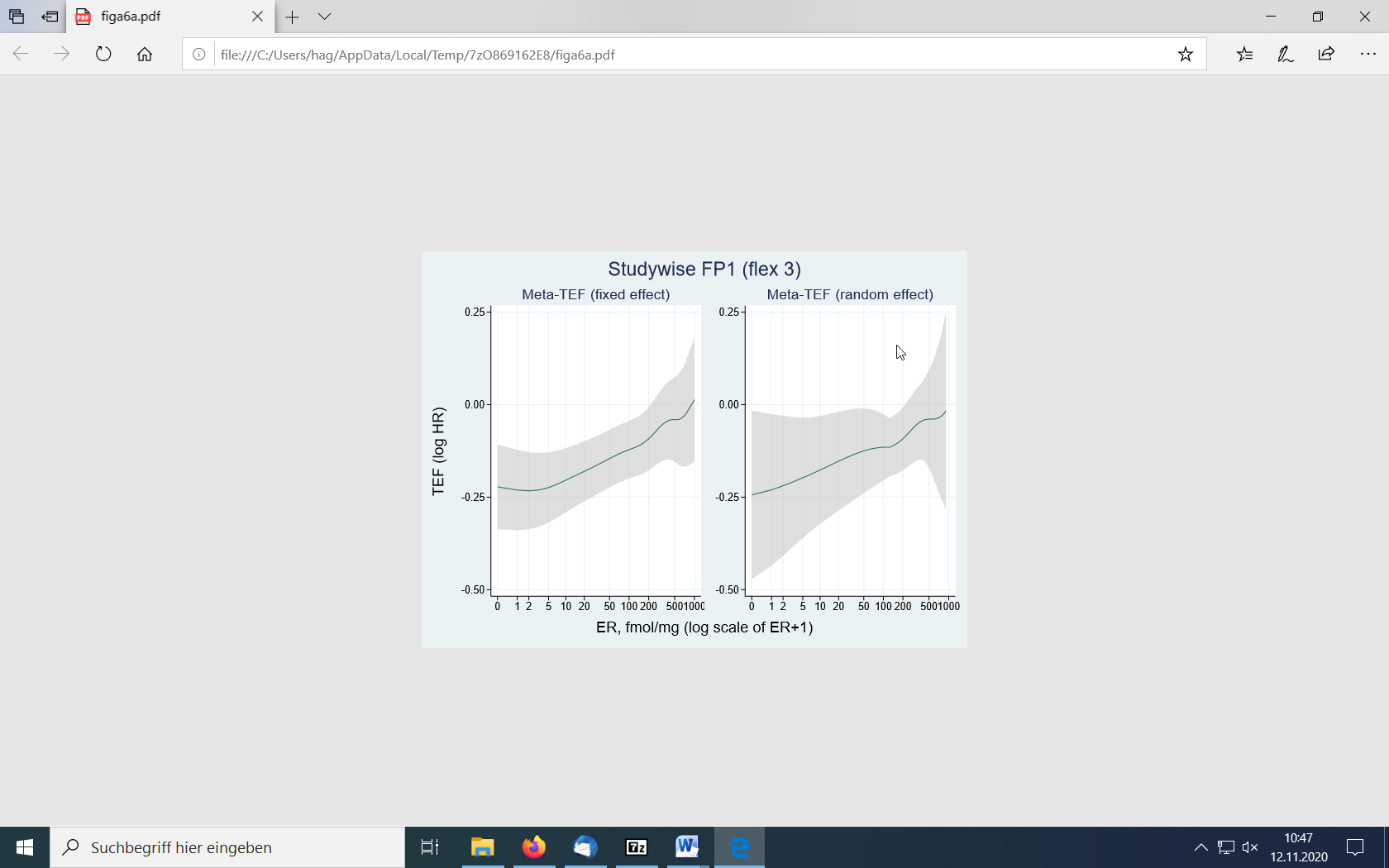

Supplement: Supplementary file 1 — Additional file 1. [file 12874_2022_1516_MOESM1_ESM.docx]
